# DRAM1 regulates apoptosis through increasing protein levels and lysosomal localization of BAX

**DOI:** 10.1038/cddis.2014.546

**Published:** 2015-01-29

**Authors:** J-J Guan, X-D Zhang, W Sun, L Qi, J-C Wu, Z-H Qin

**Affiliations:** 1 Department of Pharmacology and Laboratory of Aging and Nervous Diseases, Jiangsu Key Laboratory of Translational Research and Therapy for Neuro-Psycho-Diseases, College of Pharmaceutical Sciences, Soochow University School of Pharmaceutical Science, Suzhou, China

## Abstract

DRAM1 (*DNA damage-regulated autophagy modulator 1*) is a TP53 target gene that modulates autophagy and apoptosis. We previously found that DRAM1 increased autophagy flux by promoting lysosomal acidification and protease activation. However, the molecular mechanisms by which DRAM1 regulates apoptosis are not clearly defined. Here we report a novel pathway by which DRAM1 regulates apoptosis involving BAX and lysosomes. A549 or HeLa cells were treated with the mitochondrial complex II inhibitor, 3-nitropropionic acid (3NP), or an anticancer drug, doxorubicin. Changes in the protein and mRNA levels of BAX and DRAM1 and the role of DRAM1 in BAX induction were determined. The interaction between DRAM1 and BAX and its effect on BAX degradation, BAX lysosomal localization, the release of cathepsin B and cytochrome *c* by BAX and the role of BAX in 3NP- or doxorubicin-induced cell death were studied. The results showed that BAX, a proapoptotic protein, was induced by DRAM1 in a transcription-independent manner. BAX was degraded by autophagy under basal conditions; however, its degradation was inhibited when DRAM1 expression was induced. There was a protein interaction between DRAM1 and BAX and this interaction prolonged the half-life of BAX. Furthermore, upregulated DRAM1 recruited BAX to lysosomes, leading to the release of lysosomal cathepsin B and cleavage of BID (BH3-interacting domain death agonist). BAX mediated the release of mitochondrial cytochrome *c*, activation of caspase-3 and cell death partially through the lysosome-cathepsin B-tBid pathway. These results indicate that DRAM1 regulates apoptosis by inhibiting BAX degradation. In addition to mitochondria, lysosomes may also be involved in BAX-initiated apoptosis.

## Introduction

Autophagy and apoptosis have important roles in many biological functions of cells and are involved in pathogenesis of many diseases. The mitochondria have an important role in the initiation of apoptosis by releasing proapoptotic factors and activation of caspases, whereas the lysosomes are essential for autophagy as they provide digestive enzymes for the completion of autophagic process. Autophagy promotes cell survival or cell death under basal and various stressed conditions through its autophagic pathway or crosstalk to apoptosis.^1, 2^ The interaction between the autophagic protein beclin1 and the antiapoptotic protein Bcl-2 has defined the first molecular pathway of crosstalk between autophagy and apoptosis.^3^ DRAM1 (*DNA damage-regulated autophagy modulator 1*) is a TP53 target gene coding a lysosomal membrane protein and has an essential role in TP53-mediated autophagy activation and apoptosis.^4, 5, 6^ We have previously reported that DRAM1 was involved in the mitochondrial complex II inhibitor (3-nitropropionic acid (3NP))-induced autophagy activation and cell death.^7^ We have also characterized a mechanism by which DRAM1 stimulates autophagy flux by enhancing vacuole ATPase activity and lysosomal acidification.^8^ These studies suggest that DRAM1 regulates autophagy partially through lysosomes. However, the regulation of apoptosis by DRAM1 remains to be elucidated.

The classical apoptosis is triggered by the translocation of proapoptotic protein BAX from the cytosol to the mitochondria and induced by the release of cytochrome *c*,^9, 10^ which leads to the activation of procaspase-9 and -3. The induction of BAX has been found in the apoptotic process in response to physiological and pathological stimuli.^11, 12^ The upregulation of BAX protein was achieved by increasing its transcription or decreasing its degradation. Although ubiquitin-proteasome system (UPS) was reportedly involved in the degradation of BAX,^13^ other mechanisms were also apparently involved in the degradation of BAX.^14, 15^ Whether an autophagy-lysosomal pathway (ALP) has a role in the degradation of BAX remains to be clarified.

On the other hand, the release of cytochrome *c* from the mitochondria is also triggered by truncated BH3-interacting domain death agonist (tBID). BID is normally present in the cytosol and cleaved by activated caspase-8 or the lysosomal cathepsin B to form tBID (carboxy-terminal region of BID).^16, 17, 18^ tBID relocalizes to the mitochondria from the cytosol to release cytochrome *c*.^19^ Some investigators suggest that members of the Bcl-2 family proteins that mediate permeabilization of mitochondrial membranes may also be involved in the permeabilization of lysosomal membranes. For example, BAX was reported to increase the permeability of lysosomes,^20, 21^ but some essential evidence is still missing. We speculated that DRAM1 might regulate apoptosis through BAX and lysosomes.

In the present study, we sought to investigate if DRAM1 regulated BAX levels and if DRAM1 would recruit BAX to lysosomes through a protein–protein interaction. The recruitment of BAX to lysosomes would release lysosomal cathepsin B to cleave BID. We found that DRAM1 inhibited BAX autophagic degradation and increased BAX lysosomal localization under stress conditions, leading to lysosomal permeabilization, cathepsin B release, BID cleavage and activation of mitochondrial apoptotic pathway.

## Results

### DRAM1 increases BAX protein levels independent of transcription

**Figure 1:**
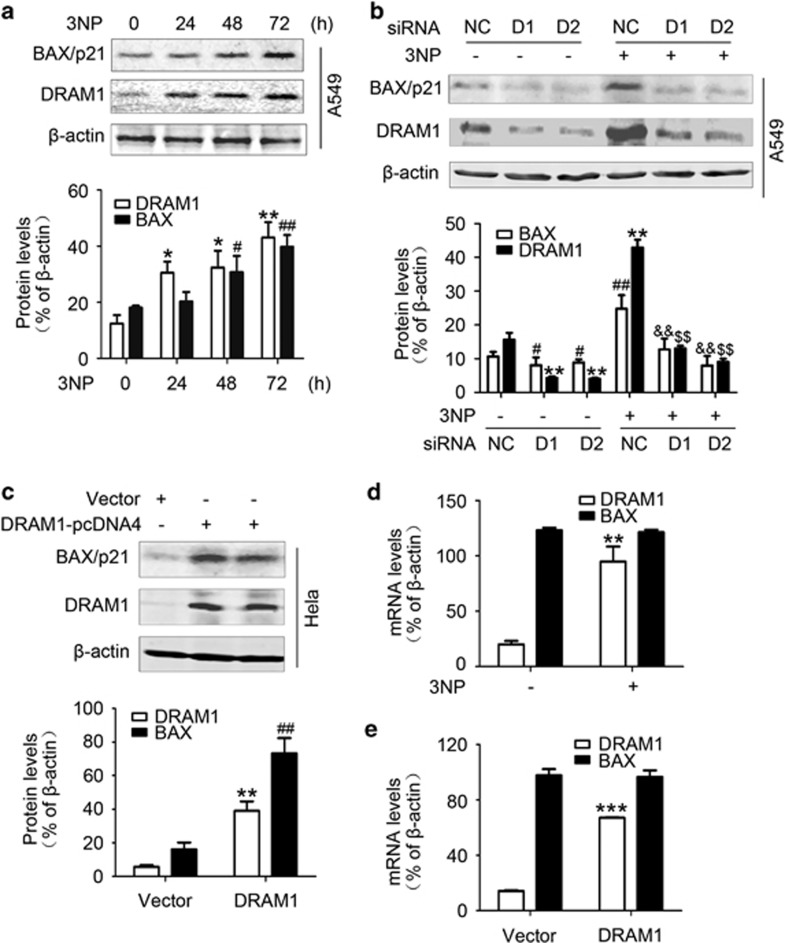
DRAM1 increases BAX protein levels independent of transcription. (**a**) A549 cells were treated with 3NP (500 *μ*M) and harvested 24, 48 and 72 h later. Bars represent mean±S.E.; *n*=4; **P<0.01 *versus* 0 h; ^##^*P*<0.01 *versus* 0 h. (**b**) A549 cells were transfected with DRAM1 small interfering RNA (siRNA) or a non-silencing siRNA for 24 h. Cells were then treated with or without 3NP (500 *μ*M) for another 24 h. Bars represent mean±SE; *n*=4; **P<0.01 *versus* NC; ^#^*P*<0.05; ^##^*P*<0.01 *versus* NC; ^$$^*P*<0.01 *versus* NC+3NP; ^&&^*P*<0.01 *versus* NC+3NP. (**c**) HeLa cells were transfected with a vector or DRAM1 plasmid for 48 h. Bars represent mean±S.E.; *n*=4; **P<0.01 *versus* vector; ^##^*P*<0.01 *versus* vector. (**d**) HeLa cells were treated with 3NP (500 *μ*M) for 48 h. RNAs were isolated and amplified with qRT-PCR. Bars represent mean±S.E.; *n*=4; **P<0.01 *versus* control. (**e**) HeLa cells were transfected with a vector or DRAM1 plasmid for 48 h. RNAs were isolated and amplified with qRT-PCR. Bars represent mean±S.E.; *n*=4; ***P<0.001 *versus* vector. NC, negative control

To determine if DRAM1 expression was involved in the regulation of apoptosis, the induction of proapoptotic protein BAX was investigated in A549 and HeLa cells. The results demonstrated that 3NP induced a significant increase in the protein levels of DRAM1 and BAX in A549 cells (Figure 1a) and HeLa cells (Supplementary Figure 1a). The upregulation of DRAM1 and BAX was also found in A549 and HeLa cells after treatment with doxorubicin (Supplementary Figures 1b and c). To evaluate whether elevation of BAX was regulated by DRAM1, the present study examined the effects of DRAM1 siRNA on 3NP- and doxorubicin-induced changes in BAX protein levels. It was shown that silencing of DRAM1 expression markedly reduced basal levels and 3NP- (Figure 1b) and doxorubicin-induced (Supplementary Figure 1d) upregulation of BAX in A549 cells. In addition, BAX induction was also observed in HeLa cells transfected with DRAM1-pcDNA4 (Figure 1c), suggesting that DRAM1 could regulate BAX protein levels. On the other hand, overexpression of BAX had no effect on DRAM1 protein levels (Supplementary Figure 1e).

To test if this BAX upregulation was dependent on transcription, we isolated DRAM1 and BAX RNAs from HeLa cells treated with 3NP or transfected with DRAM1-pcDNA4. Quantitative real-time RT-PCR (qRT-PCR) amplification of these RNAs revealed that upregulation or overexpression of DRAM1 did not increase BAX mRNA levels (Figures 1d and e), suggesting that the increase in BAX protein levels was independent of transcription.

### DRAM1 interacts with BAX and prevents it from autophagic degradation

**Figure 2:**
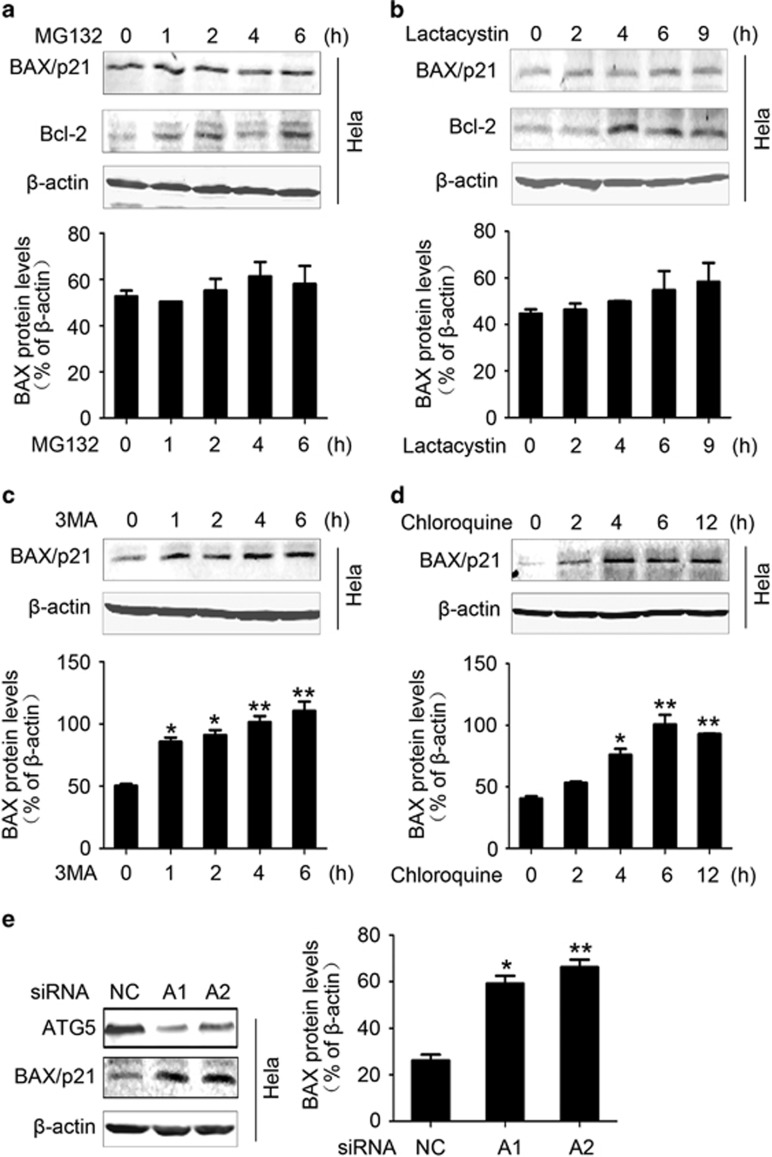
BAX is degraded by autophagy and DRAM1 interferes with BAX degradation. (**a** and **b**) Cells were treated with UPS inhibitor MG132 (40 *μ*M) for 1, 2, 4 and 6 h or lactacystin (4 *μ*M) for 2, 4, 6, and 9 h. The cell lysates were subjected to immunoblotting. *β*-Actin served as a loading control and Bcl-2 served as a positive control. Bars represent mean±S.E.; *n*=3. (**c**) HeLa cells were treated with an autophagy inhibitor 3MA (1.5 mg/ml) for 1, 2, 4 and 6 h. Bars represent mean±S.E.; *n*=4; *P<0.05; **P<0.01 *versus* 0 h. (**d**) HeLa cells were treated with an autophagy inhibitor chloroquine (20 *μ*M) for 2, 4, 6 and 12 h. Bars represent mean±S.E.; *n*=4; *P<0.05; **P<0.01 *versus* 0 h. (**e**) Cells were transfected with Atg5 small interfering RNA (siRNA) for 48 h to inhibit autophagy. Bars represent mean±S.E.; *n*=4; *P<0.05; **P<0.01 *versus* NC. NC, negative control; 3MA, 3-methyladenine

As the BAX upregulation was independent of transcription, we reasoned that DRAM1 might affect BAX degradation. To examine the involvement of UPS and ALP in the degradation of BAX, HeLa cells were treated with MG132 (40 *μ*M) for 0, 1, 2, 4 and 6 h or lactacystin for 0, 2, 4, 6 and 9 h. The data showed that inhibition of UPS did not significantly increase the protein levels of BAX, whereas it increased the levels of Bcl-2, which is a recognized substrate of UPS (Figures 2a and b).^22, 23^ The inhibition of proteasome activity by MG132 was also confirmed with a robust accumulation of ubiquitinated proteins in HeLa cells 4 h after MG132 (40 *μ*M) treatment (Supplementary Figure 2a). Next, we examined if BAX can be degraded through ALP. To inhibit autophagy activity, we treated HeLa cells with the ALP inhibitors chloroquine (20 *μ*M) and bafilomycin (100 nM) for 6 h. Both chloroquine and bafilomycin increased the accumulation of p62 and LC3, indicating that autophagy flux was blocked (Supplementary Figure 2b). Next, HeLa cells were treated with 3MA for 0, 1, 2, 4 and 6 h or chloroquine for 0, 2, 4, 6 and 12 h. The results showed a progressive increase in BAX protein levels (Figures 2c and d). The autophagic degradation of BAX was also confirmed in A549 and HCT116 cells (Supplementary Figures 2c and f). To further confirm that ALP did have a role in BAX degradation, the effect of Atg5 siRNA on BAX protein level was examined. Atg5 is required for autophagy in almost all contexts including basal autophagy.^24, 25^ Two siRNAs that have been previously validated were used to inhibit autophagy in the present study.^3^ Results showed an increase in BAX protein level when autophagy was blocked by Atg5 siRNA (Figure 2e).

It is expected that stimulation of autophagy would increase the degradation of BAX. Thus, autophagy was induced with rapamycin in HeLa cells.^26, 27^ However, data showed a progressive increase in BAX protein levels (Supplementary Figure 3a). It was found that the protein level of DRAM1 was also upregulated after treatment with rapamycin, indicating that mTOR signaling had an impact on DRAM1 expression. As the increase in BAX was dependent on DRAM1, we reasoned that DRAM1 may affect BAX degradation after rapamycin treatment. Thus, we tested if autophagy activation would accelerate the degradation of BAX in the absence of DRAM1 upregulation. The data showed a gradual decrease in BAX protein levels after rapamycin treatment in DRAM1 knockdown cells (Supplementary Figure 3b). Autophagy was also induced by the expression of exogenous DRAM1 with DRAM1-pcDNA4. The data showed that overexpression of DRAM1 increased BAX protein levels. However, autophagy inhibitors did not result in a higher BAX protein levels under DRAM1 overexpression condition (Supplementary Figures 3c and d), indicating that under different basal conditions, DRAM1-driven autophagy activation is unable to accelerate BAX degradation.

**Figure 3:**
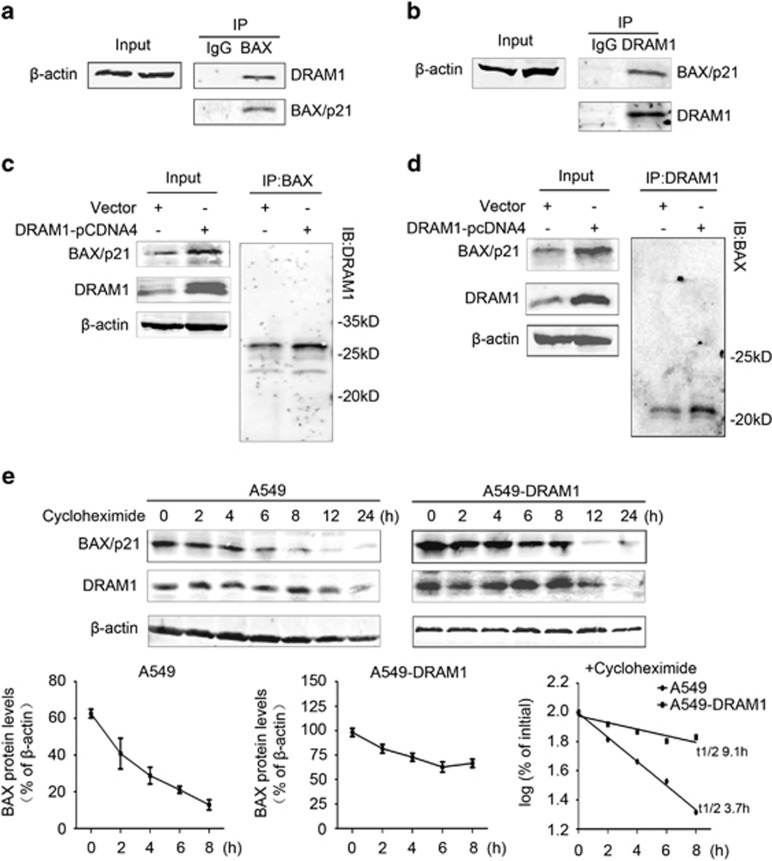
DRAM1 interacts with BAX. (**a**) Cell lysates were immunoprecipitated (IP) with an anti-BAX antibody and then immunoblotted (IB) against DRAM1. (**b**) Cell lysates were IP with an anti-DRAM1 antibody and then IB against BAX. (**c**) Cell lysates from cells transfected with vector or DRAM1-pcDNA4 were IP with an anti-BAX antibody and then IB against DRAM1. (**d**) Cell lysates as mentioned in panel c were IP with an anti-DRAM1 antibody and then IB against BAX. (**e**) The half-life of BAX was prolonged in DRAM1-overexpressing cells. A549 cells and those stably transfected with DRAM1 were treated with cycloheximide (50 *μ*g/ml). Cells were harvested at 0, 2, 4, 6, 8, 12 and 24 h after treatment. Bars represent mean±S.E.; *n*=3. The density of immunoreactivity for each band was measured and normalized to the density at *t*=0 (100%). The log10 of the percentage of density was plotted *versus* time, and the *t*_1/2_ was calculated from the log 10 of 50%

As DRAM1 increases autophagy flux, delays in BAX degradation by DRAM1 need to be addressed. One possibility is that DRAM1 may interact with BAX, thus preventing it from being targeted for lysosomal degradation. Co-immunoprecipitation revealed that DRAM1 did associate with BAX under basal conditions (Figures 3a and b). More importantly, co-immunoprecipitation of BAX was significantly increased in DRAM1-overexpressing cells (Figures 3c and d). A GST pull-down study also demonstrated that DRAM1 could bind to BAX (Supplementary Figure 4a). We next determined if DRAM1-BAX interaction would affect the association between Bcl-2 and BAX. Co-immunoprecipitation revealed that overexpression of DRAM1 increased the association between BAX and DRAM1, whereas it decreased the association between BAX and Bcl-2 (Supplementary Figure 4b). To study if starvation-induced autophagy activation also alters BAX/DRAM1 interaction, we transferred HeLa cells to serum-free medium for 4 h. The data showed that starvation increased the association between BAX and DRAM1 (Supplementary Figure 4c). These results suggest that DRAM1 interacts with BAX, and overexpression of DRAM1 results in an increase in its association with BAX.

To confirm stabilization of BAX by DRAM1, A549 cells and A549 stably expressing DRAM1 were treated with cycloheximide for 0, 2, 4, 6, 8, 12 and 24 h to determine the half-life of BAX. The data showed that cycloheximide caused a quick decline in BAX protein levels in A549 cells. A higher level of BAX in DRAM1-overexpressing cells than in normal cells was observed 2, 4, 6 and 8 h after cycloheximide treatment (Figure 3e). BAX protein levels were markedly decreased in DRAM1-overexpressing cells 12 h after cycloheximide treatment, the time when DRAM1 started to decline. Using BAX protein levels from 0 to 8 h after cycloheximide treatment, the deduced half-life of BAX was 3.7 h in normal cells and 9.1 h in cells with the overexpression of DRAM1 (Figure 3e). These data indicate that BAX is targeted by autophagy under basal conditions, but its degradation is inhibited when DRAM1 is induced.

### DRAM1 recruits BAX to lysosomes

**Figure 4:**
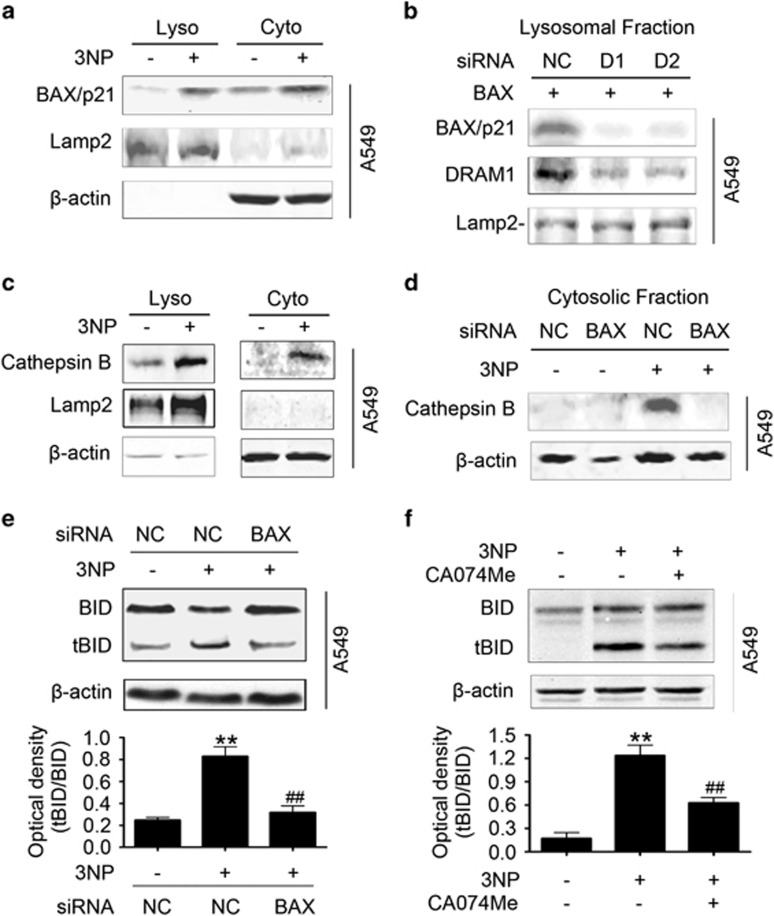
BAX translocates to lysosome, releases cathepsin B and activates the BID cleavage. (**a**) A549 cells were treated with 3NP (500 *μ*M) for 48 h. The cytoplasmic (Cyto) and lysosomal (Lyso) fractions were fractionated and then subjected to sodium dodecyl sulfate-polyacrylamide gel electrophoresis (SDS-PAGE). (**b**) Lysosomes from cells transfected with or without DRAM1 small interfering RNA (siRNA) were fractionated and then incubated with BAX protein at 37 °C for 2 h. The lysosomal fractions were subjected to SDS-PAGE. (**c**) A549 cells were treated with 3NP (500 *μ*M) for 48 h. The Cyto and Lyso were fractionated and then subjected to SDS-PAGE. (**d**) A549 cells were transfected with BAX siRNA or a non-silencing siRNA for 24 h and then treated with 3NP (500 *μ*M) for another 24 h; the cytoplasm was fractionated for western blot analysis. (**e**) A549 cells were transfected with BAX siRNA or a non-silencing siRNA for 24 h and then treated with or without 3NP (500 *μ*M) for another 24 h. Bars represent mean±S.E.; *n*=4; **P<0.01 *versus* NC; ^##^*P*<0.01 *versus* 3NP+NC. (**f**) A549 cells were treated with 3NP (500 *μ*M) and the cathepsin B inhibitor for 24 h. Bars represent mean±S.E.; *n*=4; **P<0.01 *versus* control; ^##^*P*<0.01 *versus* 3NP. NC, negative control

As DRAM1 is a lysosomal protein and DRAM1 interacts with BAX as demonstrated by immunoprecipitation and GST pulldown, we speculated that DRAM1 could recruit BAX to lysosomes. A549 cells transiently transfected with BAX-EGFP were treated with or without 3NP, and the lysosomal localization of BAX was examined using LysoTracker. The results showed partial colocalization of BAX-EGFP and LysoTracker after 3NP treatment (Supplementary Figure 5a). Western blot analysis showed that the BAX protein level was increased in the lysosome-enriched fraction after 3NP treatment (Figure 4a), indicating that 3NP may induce the translocation of BAX to lysosomes. To determine the translocation of BAX from the cytoplasm to mitochondria, we isolated the mitochondria and lysosomes with the overexpression of DRAM1 after 3NP treatment. The data showed that 3NP treatment alone induced translocation of BAX to the mitochondria and lysosomes. However, with the overexpression of DRAM1, 3NP caused more BAX translocation to lysosomes *versus* mitochondria (Supplementary Figure 5b). To examine if this lysosomal localization of BAX was dependent on DRAM1, the colocalization of BAX and LysoTracker was then observed in the absence of DRAM1 induction. The result showed that DRAM1 siRNA partially inhibited the lysosomal translocation of BAX (Supplementary Figure 5a). Recombinant BAX protein was used to incubate lysosomes isolated from control cells and cells transfected with DRAM1 siRNA. Western blot analysis was performed to examine the protein levels of recombinant BAX, which associated with lysosomes after incubation. Results showed that less recombinant BAX protein was recruited to lysosomes in DRAM1-downregulated cells than in normal cells (Figure 4b), indicating the lysosomal localization of BAX is dependent on DRAM1.

### BAX causes the release of lysosomal proteases and cleavage of BID

Data from multiple groups have shown that BAX can be recruited to the mitochondria and form channels with other proapoptotic proteins, and can also participate in the release of cytochrome *c*.^9, 10^ Although BAX was also proposed to permeabilize lysosomal membranes, the underlying mechanism was missing. In the present study, immunofluorescent technique was used to evaluate the release of cathepsin B. Cells were treated with or without 3NP for 48 h, and then the colocalization of cathepsin B (green) and Lamp2 (red) was assessed. Results showed that 3NP increased the diffusion of cathepsin B in the cytosol, indicating the release of cathepsin B from lysosomes (Supplementary Figure 5c). Cells were treated with or without 3NP for 48 h and then cell lysates were fractionated into the cytosolic and the lysosomal fractions. Western blot analysis showed that cathepsin B was released from lysosomes into the cytosol after treatment with 3NP (Figure 4c). To test if BAX has a role in the release of cathepsin B from lysosomes, BAX was knocked down with siRNA (Supplementary Figure 6a). The *N*-acetyl*β*-d-glucosaminidase (NAG), a symbolic enzyme of lysosome was used to test if the integrity of lysosomal membrane was disrupted when BAX was recruited to lysosomes.^28^ The data showed a leakage of NAG from lysosomes after 3NP treatment, and this was markedly reduced by silencing BAX expression (Supplementary Figure 6b). The results also showed that knockdown of BAX inhibited 3NP, which induced the increase in the cytosolic cathepsin B (Figure 4d). Lysosomes were isolated and then incubated with or without recombinant BAX protein at 37 °C for 2 h. After incubation, lysosomal sediments and supernatants were collected for analysis. The data showed that cathepsin B was released from the lysosomes after incubation with recombinant BAX (Supplementary Figure 6c), indicating that BAX can permeabilize the lysosomal membranes and the release of cathepsin B.

Lysosomes are involved in an apoptotic pathway through cathepsin B-tBID-caspase-3 pathway.^17, 18^ To assess if BAX has a role in 3NP-induced apoptosis partially through lysosomes by regulating cleavage of BID, BAX siRNA was infected into A549 cells and the effects of BAX siRNA on 3NP-induced cleavage of BID was examined. The data showed that 3NP induced BID cleavage, and BAX silencing markedly reduced 3NP-induced tBID production (Figure 4e). 3NP-induced cleavage of BID was also inhibited by the cathepsin B inhibitor (Figure 4f), whereas the caspase-8 inhibitor had no effect (Supplementary Figure 6d), indicating that 3NP-induced cleavage of BID was mediated by the lysosomal BAX and cathepsin B. Another classical apoptotic stimulus etoposide also induced cleavage of BID.^29^ The results demonstrated that etoposide induced a significant increase in the protein levels of DRAM1 and BAX in A549 cells from 12 to 24 h (Supplementary Figure 6e), whereas caspase-8 inhibitor decreased etoposide-induced cleavage of BID (Supplementary Figure 6f), indicating that etoposide-induced cleavage of BID was caspase-dependent.

### BAX mediates the release of cytochrome *c* and apoptosis

**Figure 5:**
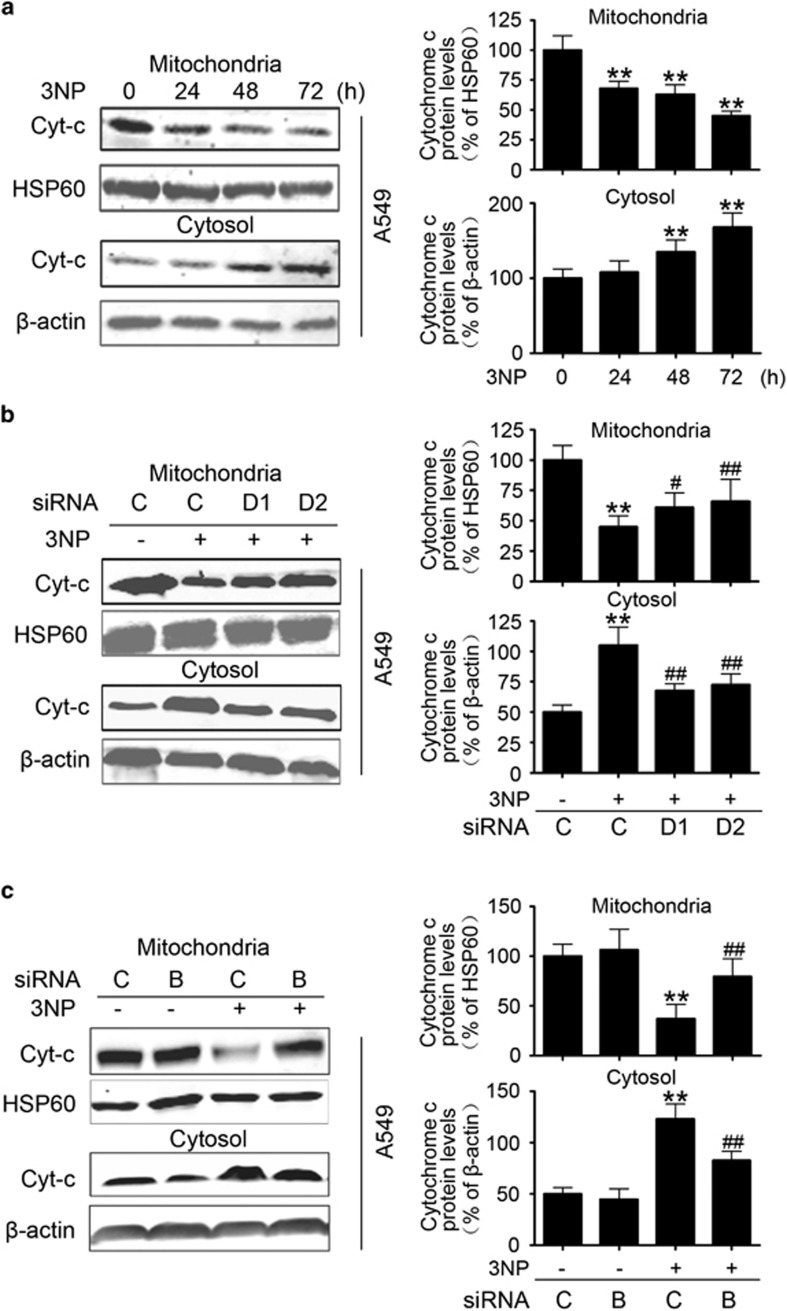
BAX mediates the proapoptotic role of DRAM1. (**a**) A549 cells were treated with 3NP (500 *μ*M) and harvested 24, 48 and 72 h later for the preparation of mitochondrial and cytosolic fractions. Bars represent mean±S.E.; *n*=3; **P<0.01 *versus* 0h. (**b**) A549 cells were transfected with DRAM1 small interfering RNA (siRNA) or non-silencing (Non-sil) siRNA for 24 h and then treated with 3NP (500 *μ*M) for 24 h. Bars represent mean±S.E.; *n*=3; **P<0.01 *versus* NC; ^#^*P*<0.05; ^##^*P*<0.01 *versus* 3NP+NC. (**c**) A549 cells were transfected with BAX siRNA or Non-sil siRNA for 24 h and then treated with 3NP (500 *μ*M) for 24 h. Bars represent mean±S.E.; *n*=3; **P<0.01 *versus* NC; ^##^*P*<0.01 *versus* 3NP+NC. NC, negative control

**Figure 6:**
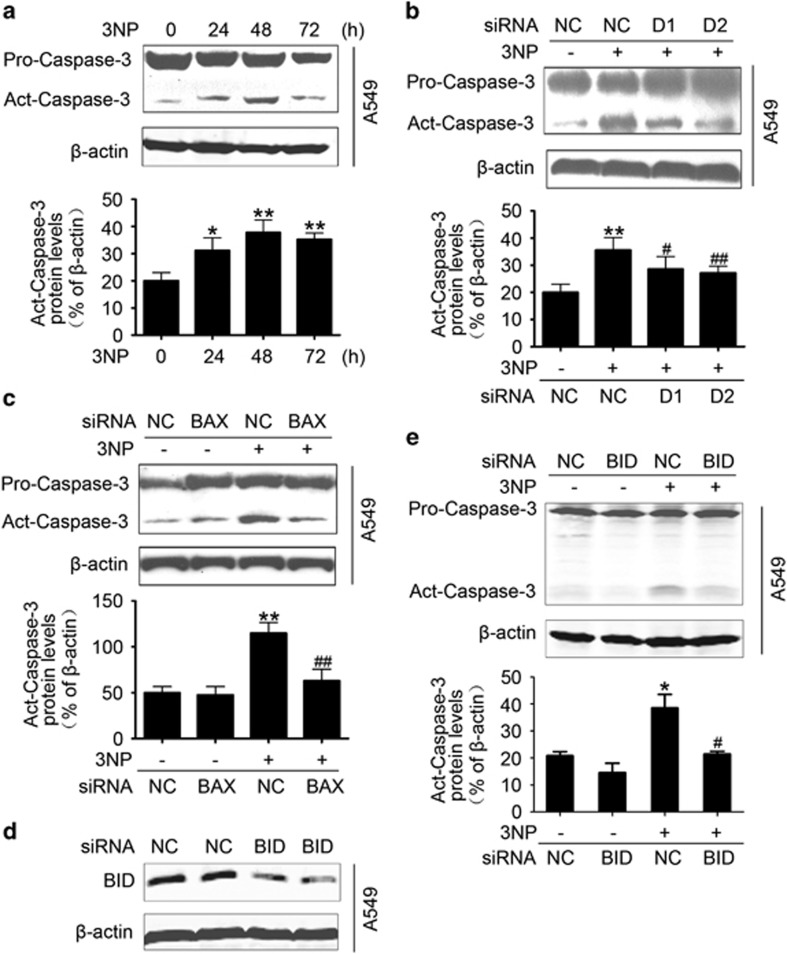
BAX mediates proapoptotic role of DRAM1 partially through tBID. (**a**) Immunoblot analysis of the activation of caspase-3. A549 cells were treated with 3NP (500 *μ*M) for 24, 48 and 72 h; the activation of caspase-3 was determined by immunoblotting. Bars represent mean±S.E.; *n*=3; *P<0.05, **P<0.01 *versus* 0 h. (**b**) A549 cells were transfected with DRAM1 small interfering RNA (siRNA) or non-silencing (Non-sil) siRNA for 24 h and then treated with 3NP (500 *μ*M) for 24 h. Bars represent mean±S.E.; *n*=3, **P<0.01 *versus* NC; ^#^*P*<0.05, ^##^*P*<0.01 *versus* 3NP+NC. (**c**) A549 cells were transfected with BAX siRNA or Non-sil siRNA for 24 h and then treated with 3NP (500 *μ*M) for 24 h. Bars represent mean±S.E.; *n*=3, **P<0.01 *versus* NC; ^##^*P*<0.01 *versus* 3NP+NC. (**d**) Efficiency of siRNA-mediated downregulation of BID. (**e**) A549 cells were transfected with BID siRNA or Non-sil siRNA for 24 h and then treated with 3NP (500 *μ*M) for another 24 h. Active caspase-3 was determined by immunoblotting. Bars represent mean±S.E.; *n*=3; *P<0.05 *versus* NC, ^#^*P*<0.05 *versus* NC+3NP. NC, negative control

**Figure 7:**
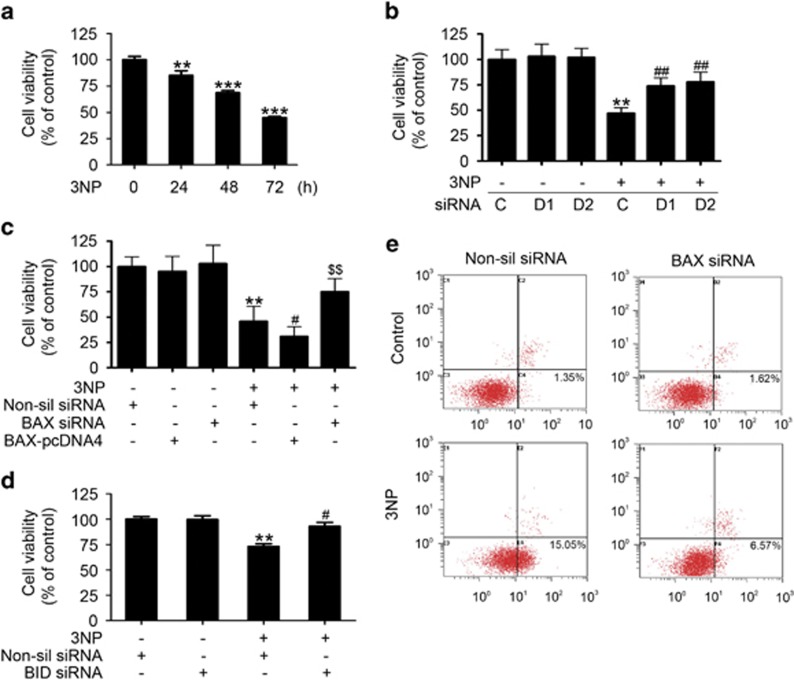
DRAM1 regulates cell death through BAX. (**a**) A549 cells were treated with 3NP (500 *μ*M) for 24, 48 and 72 h, and the cell viability was evaluated. Bars represent mean±S.E.; *n*=4; **P<0.01; ***P<0.001 *versus* 0 h. (**b**) Cell viability was evaluated after transfection of cells with DRAM1 small interfering RNA (siRNA) in the presence or absence of 3NP (500 *μ*M). Bars represent mean±S.E.; *n*=4; **P<0.01 *versus* NC; ^##^*P*<0.01 *versus* NC+3NP. (**c**) Cell viability was evaluated after transfection of cells with BAX-pcDNA4 or BAX siRNA in the presence or absence of 3NP (500 *μ*M). Bars represent mean±S.E.; *n*=4; **P<0.01 *versus* NC; ^#^*P*<0.05 *versus* NC+3NP; ^$$^*P*<0.01 *versus* NC+3NP. (**d**) A549 cells were transfected with BID siRNA for 24 h and then treated with 3NP (500 *μ*M) for another 24 h. Bars represent mean±S.E.; *n*=3; **P<0.01 *versus* NC; ^#^*P*<0.05 *versus* 3NP+non-silencing (Non-sil) siRNA. (**e**) A549 cells were transfected with BAX siRNA or Non-sil siRNA for 24 h and then treated with 3NP (500 *μ*M) for another 24 h. After treatment, apoptotic cells were determined with FACS after Annexin V-FITC and PI staining. NC, Negative control. NC, negative control

Given the role of BAX and BID in apoptosis,^17, 30^ the time course of 3NP-induced changes in cytochrome *c* release and caspase-3 activation in A549 cells was determined 24 to 72 h after 3NP treatment. The data showed the release of cytochrome *c* from the mitochondria to the cytosol (Figure 5a) and the activation of caspase-3 after 3NP treatment (Figure 6a). Here we demonstrate that overexpression of DRAM1 alone did not induce the activation of caspase-3 (Supplementary Figure 7a). The fluorescence-activated cell sorting (FACS) data also showed that overexpression of DRAM1 did not induce apoptosis (Supplementary Figure 7b); however, silencing DRAM1 expression markedly reduced 3NP, which induced the release of cytochrome *c* from the mitochondria (Figure 5b) and the activation of caspase-3 (Figure 6b). Next, we showed that knockdown of DRAM1 inhibited the 3NP-induced apoptosis (Supplementary Figure 7c), whereas overexpression of DRAM1 decreased cell viability in the presence of 3NP (Supplementary Figure 7d). Our studies also showed that knockdown of Atg5 also increased cell viability following 3NP treatment (Supplementary Figure 7e). Moreover, this study showed that 3NP induced the release of cytochrome *c*, and the activation of caspase-3 was significantly attenuated after knockdown of BAX with siRNA (Figure 5c and Figure 6c), suggesting that DRAM1-dependent BAX upregulation is involved in 3NP-induced mitochondrial apoptotic pathway. To confirm if BID cleavage contributes to 3NP-induced apoptosis, BID siRNAs, which have been previously validated,^31^ were used to silence BID (Figure 6d). The data showed that 3NP-induced caspase-3 activation was partially inhibited by BID siRNA (Figure 6e). 3NP induced robust cell death in A549 cells (Figure 7a). Knockdown of DRAM1 significantly reduced 3NP-induced cell death (Figure 7b). Results also showed that overexpression of BAX increased, while silencing BAX significantly reduced, 3NP-induced cell death (Figure 7c). To test the contribution of tBID in 3NP-induced cell death, cell viability was assessed after cells were treated with BID siRNA and the data showed that 3NP-induced cell death was partially inhibited by BID siRNA (Figure 7d). After staining cells with annexin V-FITC and PI, FACS was used to determine the effects of BAX siRNA on doxorubicin-induced apoptotic cells death. After treatment with doxorubicin, the percentage of apoptotic cells increased from 1.35 to 15.05% in the control culture. The percentage of apoptotic cells decreased to 6.57% when BAX expression was inhibited with siRNA (Figure 7e), suggesting that DRAM1-dependent BAX upregulation is involved in 3NP-induced mitochondrial apoptotic pathway.

## Discussion

The molecular pathways that lead to apoptosis are relatively well defined, but the molecular machinery that was involved in autophagic cell death has not been defined clearly. However, it is generally accepted that there is a crosstalk between autophagy and apoptosis. The crosstalk between apoptosis and autophagy was first described in 2005. Researchers discovered that Bcl-2 could not only act as antiapoptotic protein but could also inhibit autophagy via its inhibitory interaction with beclin1.^3^ After that, a regulator of autophagy, UVRAG, was reported to inhibit apoptosis by blocking the activation and mitochondrial translocation of BAX.^32^ Other investigators found that the autophagy protein Atg12 could associate with Bcl-2 family members to promote mitochondrial apoptosis.^33^ All these findings indicate that there is a complex interplay between autophagy and apoptosis.

**Figure 8:**
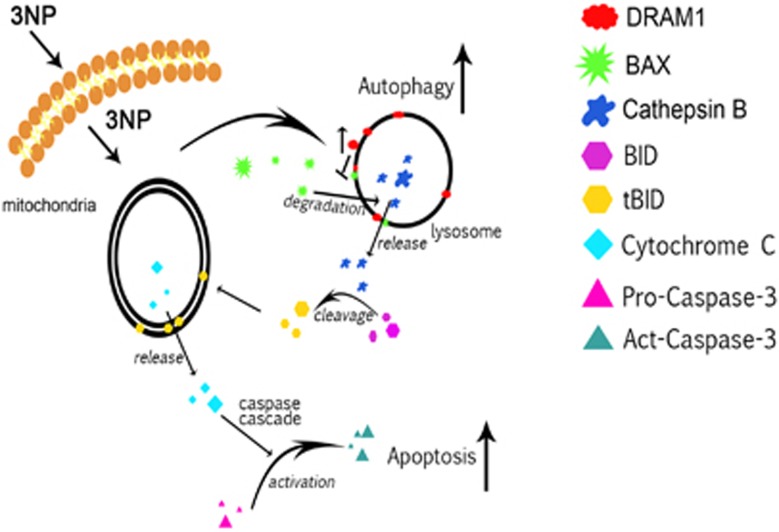
Proposed model for the action of DRAM1 in 3NP- and doxorubicin-induced autophagy and apoptosis

*DRAM1* was first reported as a TP53 target gene that modulated autophagy. Although DRAM1 cannot induce apoptosis by itself, it is a critical component of TP53-induced apoptosis.^4, 5, 34^ In previous studies, we reported that DRAM1 promoted autophagy flux through lysosomes. Here we provided evidence that DRAM1 can regulate apoptosis through lysosomes (Figure 8).

The significance of our findings is several folds. First, we demonstrated that DRAM1 increased the protein levels of BAX independent of transcription. 3NP increased protein levels of DRAM1 and BAX in several cell lines, whereas knockdown of DRAM1 inhibited 3NP- and doxorubicin-induced upregulation of BAX. DRAM1 increased BAX levels but had no effect on BAX mRNA levels. This positive role of DRAM1 in BAX protein levels provides a new molecular link between DRAM1 and apoptotic signaling.

Second, we demonstrated that BAX was degraded by autophagy and DRAM1 inhibited autophagic degradation of BAX, underscoring the molecular mechanism by which DRAM1 increases the protein levels of BAX. Although data from other groups showed that BAX could be ubiquitinated,^13, 35, 36^ the protein level of BAX did not increase when UPS was inhibited with two small-molecule inhibitors. One possible reason may be because different isoforms of BAX were examined. Different from reported studies,^13, 37^ in the present study only BAX*α* (21KD) was examined. Our results were in agreement with other studies showing that MG132 did not increase the protein levels of BAX*α*, while it induced the accumulation of protein levels of BAXβ (24 KD), a constitutively active human BAX isoform.^14, 38^ We here proved that BAX, or at least a portion of BAX, was degraded by ALP by means of pharmacological and molecular manipulations. Interestingly, the protein levels of BAX did not decrease when autophagy was activated by rapamycin because of an upregulation of DRAM1 caused by mTOR inhibition. The data from other groups showed that TP53 could modulate mTOR signaling to activate autophagy.^39, 40^ Our present findings demonstrated that inhibition of mTOR by rapamycin increased autophagy activity and DRAM1 expression. This may suggest that mTOR inhibition is upstream of DRAM1 induction.

Third, the novelty of the present study is that we demonstrated the protein interaction between DRAM1 and BAX. Several previous reports showed that some autophagy-related proteins could take part in signaling pathways by interacting with other signaling proteins. For example, beclin1, an evolutionarily conserved autophagy protein, was reported to interact with Bcl-2, and this interaction inhibits beclin1-dependent autophagy.^3^ After that, a regulator of autophagy, UVRAG, was reported to inhibit apoptosis by blocking the activation and mitochondrial translocation of BAX.^32^ Other investigators found that the autophagy protein Atg12 could associate with Bcl-2 family members to promote mitochondrial apoptosis.^33^ All these reports lead us to test whether DRAM1 can interact with BAX to make BAX protein more stable. In this study, we found that DRAM1 could bind to BAX to prolong the half-life of BAX. Both immunoprecipitation and *in vitro* pull-down assay showed that DRAM1 interacted with BAX, and overexpression of DRAM1 results in an increase in its association with BAX. The increased association between DRAM1 and BAX affected the interaction between BAX and Bcl-2.

Finally, we demonstrated that, in addition to mitochondrial localization of BAX, DRAM1 could recruit BAX to lysosomes to activate the lysosome-initiated apoptotic pathway. The classical mitochondrial apoptotic pathway is triggered by BAX or tBID mitochondrial translocation. The present study found that 3NP induced an upregulation of BAX and DRAM1, leading to an increased BAX lysosomal localization in a DRAM1-dependent manner. It has been suggested that BAX can be distributed to lysosomes to affect lysosome permeability and cathepsin release,^21, 41^ but detailed evidence has not been provided. The present study thus examined the effects of BAX on lysosomal protease translocation and cleavage of BID. We found that 3NP increased tBID levels through cathepsin B. Similar to BAX, tBID can translocate to the mitochondria to induce the release of cytochrome *c*.^17, 22^ Knockdown of BAX or the cathepsin B inhibitor inhibited 3NP-induced tBID production, the release of cytochrome *c* from mitochondria and activation of caspase-3. On the other hand, etoposide, a classical apoptosis inducer through mitochondria, also induced BID cleavage, but this cleavage was caspase-dependent. Furthermore, 3NP-induced cell death was reduced by knockdown of DRAM1, BAX or BID. These data suggest that tBID produced by lysosomal recruitment of BAX and cathepsin B release contributes to the regulation of apoptosis by DRAM1.

In conclusion, the present study suggests that DRAM1 may influence apoptosis through the upregulation of BAX and BAX lysosomal translocation. Thus, this study revealed a novel mechanism of crosstalk between autophagy and apoptosis mediated by DRAM1.

## Materials and Methods

### Reagents and antibodies

MG132 and lactacystin were obtained from Calbiochem (Darmstadt, Germany; 474790 and 426100). Chloroquine, 3MA, 3NP, doxorubicin, cycloheximide and etoposide were obtained from Sigma-Aldrich China (Shanghai, China; C6628, M9281, N5636, D1515, N11534 and E1383). The antibodies used were as follows: anti-DRAM1 (Abcam, Cambridge, MA, USA; ab58807), anti-BAX (Abcam; ab5714), anti-Bcl-2 (Santa Cruz, Dallas, TX, USA; sc-7382), anti-Atg5 (Cell Signaling, Beverly, MA, USA; 2630), anti-caspase-3 (Santa Cruz; sc-271028), anti-cathepsin B (Abcam; ab49231), anti-BID (Cell Signaling; 2002), anti-Lamp2 (Santa Cruz; sc-20004), anti-cytochrome *c* (Santa Cruz; sc-7159), anti-HSP60 (Santa Cruz; sc-65568), anti-p62 (Abcam; ab96134), anti-LC3 (MBL, Nagoya, Japan; PD015 ) and anti-*β*-actin (Santa Cruz; sc-47778).

### Cell culture and cell line establishment

A549 and HeLa cell lines were purchased from Shanghai Institute of Biochemistry and Cell Biology (Shanghai, China) and were cultured in DMEM medium supplemented with 10% FBS (Hyclone, Logan, UT, USA) in a humidified incubator containing 5% CO_2_ at 37 °C.^42^ To establish a stable DRAM1-overexpressing cell line, A549 cells were infected with an empty retroviral vector as control or a retroviral-expressing DRAM1. Puromycin (6 *μ*g/ml) was added 72 h after infection. After 14 days of expansion in the presence of puromycin,^43^ the high expression of DRAM1 was determined with western blot analysis. The stable cell line was expanded and stored for future use.

### Protein knockdown and overexpression

DRAM1 was cloned into *Xho*lI and *Bam*HI sites of pcDNA4 (Invitrogen, Carlsbad, CA, USA). BAX-pcDNA4 and EGFP-BAX was a generous gift from Professor Guanghui Wang (Soochow University School of Medicine, Suzhou, China). For transfection, cells were plated in 9-cm dishes at 30% confluency, and plasmid or siRNA duplexes (200 nM) were introduced into the cells using Lipofectamine 2000 (Invitrogen) according to the manufacturer's recommendation. The siRNA target sense sequences used were as follows: DRAM1, 5′-CCACGATGTATACAAGATA-3′ and 5′-CCACGAAATCAATGGTGA-3′ Atg5, 5′-GCAACTCTGGATGGGATTG-3′ and 5′-CATCTGAGCTACCCGGATA-3′ BAX, 5′-AACATGGAGCTGCAGAGGATGATT-3′ BID, 5′-GAAGACATCATCCGGAATA-3′ and negative control, 5′-TAAGGCTATGAAGAGATAC-3′. The efficiency of knockdown-specific proteins was determined with western blot analysis 48 h after siRNA treatment.

### Quantitative real-time RT-PCR

HeLa cells were transfected with or without DRAM1-pcDNA4 for 48 h. After transfection, total RNA was prepared with RNAiso Plus (Takara, Dalian, China) and cDNA was synthesized from 1 *μ*g of RNA with M-MLV First-Strand Kit (Invitrogen). qPCR was performed with Platinum SYBR Green qPCR SuperMix-UDG (Invitrogen) with C1000 Thermal Cycler (Bio-Rad, Hercules, CA, USA). The primers used were as follows: β-actin, 5′-TCCACCACCCTGTTGCTGTA-3′ (forward) and 5′-ACCACAGTCCATGCCATCAC-3′ (reverse); DRAM1: 5′-TCAAATATCACCATTGATTTCTGT-3′ (forward) and 5′-GCCACATACGGATGGTCATCTCTG-3′ (reverse); BAX: 5′-GGACGAACTGGACAGTAACATGG-3′ (forward) and 5′-GCAAAGTAGAA-AAGGGCGACAAC-3′ (reverse). To exclude the contamination of unspecific PCR products such as primer dimers, melting-curve analysis was applied to all final PCR products after the cycling protocol. Also, the quantity of products was screened with agarose gels.

### BAX half-life assay

A549 normal cells and DRAM1-overexpressing cells were treated with cycloheximide (50 *μ*g/ml; Sigma) to inhibit further protein synthesis. Following treatment for 2, 4, 6, 8, 12 and 24 h, cells were harvested and western blot analysis was performed. The density of immunoreactivity for each protein band was measured and normalized so that the density at *t*=0 was set as 100%. The log 10 of the percentage of density was plotted *versus* time, and the *t*_1/2_ was calculated from the log 10 of 50%.^44^

### Immunoprecipitation and *in vitro* pull-down assay

For immunoprecipitation, HeLa cells were transfected with either a control/empty vector or plasmid with DRAM1 insert for 48 h before lysis. Two micrograms of protein lysates was incubated with 2 *μ*g of corresponding antibody overnight at 4 °C with end-to-end rotation, followed by incubation with IgG/IgA beads (Beyotime, Nantong, China). Beads were washed three times in 1 × PBS (2.67 mM KCl, 1.47 mM KH_2_PO_4_, 137.93 mM NaCl, 8.06 mM Na_2_HPO_4_, pH=7.4). Immunoprecipitates and starting cell lysates were subjected to standard western blot analysis.

Purified GST or BAX fusion proteins were obtained from Proteintech (Chicago, IL, USA). The G-Sepharose (GE Healthcare, Piscataway, NJ, USA) beads were washed extensively with binding buffer (1% Triton X-100, 50 mM Tris-HCl, pH 7.4, 250 mM NaCl) and were subsequently incubated with GST or BAX fusion proteins for 2 h. After centrifugation, the protein–bead complex was incubated with cell lysates for another 2 h and then pulled down. The bound proteins were subjected to SDS-PAGE and immunoblot analysis.

### Immunofluorescence microscopy

For immunofluorescence microscopic examination, A549 cells were plated on 12-mm poly-l-lysine-coated coverslips and cultured for 24 h and then cells were treated with 3NP (500 *μ*M) for 48 h or transfected with EGFP-BAX expression plasmids using Lipofectamine 2000 (Invitrogen). The culture medium was removed and the prewarmed (37 °C) medium containing LysoTracker was added after 48 h of transfection. Cells were incubated for 1 h under growth conditions and then cells were washed in PBS, fixed with 4% paraformaldehyde in PBS at 4 °C for 10 min and washed again with PBS. The coverslips were mounted on slides with mounting medium (F4680; Sigma) and were examined with a laser scanning confocal microscopy (Nikon, C1S1, Tokyo, Japan).

### Isolation of mitochondria and analysis of BAX and cytochrome *c* levels

Cells were homogenized in buffer A (250 mM mannitol, 250 mM sucrose, 1 mM EDTA, 50 mM Tris-HCl, 1 mM DTT, 1 mM PMSF, 1 mM benzamidine, 0.28 U/l apotinin, 50 mg/l leupeptin, 7 mg/l pepstain A). Homogenates were centrifuged at 1000 × *g* for 10 min and the resultant supernatant was then centrifuged at 10 000 × *g* for 30 min, and the mitochondrial pellet was resuspended in buffer B (250 mM sucrose, 1 mM EGTA, 10 mM Tris-HCl). Supernatants were separated from the mitochondria by centrifugation at 100 000 × * g* for 1 h, and the supernatant was then used to analyze cytochrome *c* in the cytosol with immunoblotting. Total mitochondrial proteins and the postmitochondrial supernatants were electrophoretically separated on 12.0% SDS-polyacrylamide gels and transferred to 0.2-*μ*m nitrocellulose membranes. The membranes were blocked with 5% (wt/vol) non-fat milk for 1 h, followed by incubation for another 1 h with mouse anti-cytochrome *c* monoclonal antibody (BD Biosciences Pharmingen, San Jose, CA, USA). The blots were then incubated with donkey anti-mouse IgG conjugated with horseradish peroxidase (Sigma). Enhanced chemoluminescent autoradiography (ECL Kit; Amersham, Arlington Heights, IL, USA) was used for the final detection. The mitochondrial preparation was also used for the determination of BAX protein levels with immunolotting.

### Isolation of lysosomes and assay of translocation of BAX and cathepsin B

The isolation of lysosomes was preformed with Lysosome Enrichment Kit for Tissue and Cultured Cells (Thermo, Rockford, IL, USA). Two hundred micrograms of A549 cells was harvested and then homogenized. Homogenates were then centrifuged at 500  × *g* for 10 min at 4 °C and the supernatants were collected. The supernatants mixed with OptiPrep Cell Separation Media were overlayed on the top of a prepared discontinuous density gradient. Samples were centrifuged at 145 000 × *g* for 2 h at 4 °C to form several bands; the lysosomal and cytoplasmic bands were removed. The lysosomal band was mixed with two to three volumes of PBS, followed by centrifugation at 18 000 × *g* for 30 min at 4 °C. The lysosomes were lysed with 2% CHAPS in PBS. The cytoplasmic and lysosomal fractions were subjected to SDS-PAGE and immunoblotted for BAX and cathepsin B.

### Assay of the integrity of lysosomal membranes

The integrity of the lysosomal membrane in cultured cells was monitored by analyzing the presence of dissociative NAG activity. Lysosomes were isolated from A549 cells treated with 3NP after transfection with non-sil RNA or BAX siRNA. The dissociative NAG and total NAG of each sample were determined with Lysosomal Membrane Integrity Kit (Genmed, Plymouth, MN, USA). Lysosomes were treated with NAG after incubation with lysates or PBS. The reaction mixture was incubated at 37 °C for 5 min and then stopped using 10 *μ*l of stop solution. The absorbance was measured at 365 nm. The integrity of lysosomal membrane was represented by the percent dissociation of NAG.

### *In vitro* assay of cathepsin B release from lysosomes

Lysosomes were isolated from A549 cells and then incubated with or without BAX protein at 37 °C for 2 h. After incubation, the supernatant and lysosomal sediment fractions were collected by centrifugation and then subjected to SDS-PAGE and immunoblotting for cathepsin B and Lamp2.

### Cell viability assay

The effect of 3NP on the viability of A549 was evaluated using a WST-1 Cell Proliferation Kit (Roche Applied Science, Basel, Switzerland) according to the kit specifications. A549 cells were plated in 96-well culture plates in a final volume of 100 *μ*l/well and were cultured in a humidified atmosphere (37 °C, 5% CO_2_). Cells were allowed to adhere to the plate surface for 24 h before being exposed to drugs. Assays were performed six times. WST-1 reagent (10 *μ*l) was added to each well and then incubated for 40 min in a humidified atmosphere (37 °C, 5% CO_2_). Quantification of the formazan dye produced by metabolically active cells was performed by measuring absorbance at 450 nm with microplate ELISA reader (ELx800; Bio-TEK Instruments, Winooski, VT, USA).

### Caspase assays

Caspase-3 and caspase-8 activities in A549 cells were measured using Caspase-Glo Assay Kit (Promega, Madison, WI, USA) for the detection of apoptosis.^45^ Briefly, DRAM1 or Atg5 knockdown cells were plated in 96-well plates and then incubated for 24 h. For caspase inhibition assay, caspase-8 inhibitor II and negative control were added 30 min before adding BIX-01294. BIX-01294 was added into the medium and then incubated for 24 h. The proluminescent substrate containing DEVD or LETD (EMD Millipore, Darmstadt, Germany), which are cleaved by caspase-3 or caspase-8, respectively, was added to each well and then incubated at room temperature for 1 h. The luminescence of each sample was measured with a microplate ELISA reader (ELx800; Bio-Tek Instruments).

### Flow cytometry

The effects of 3NP and BAX siRNA on apoptosis were evaluated using a BU-Annexin V-FITC Apoptosis Detection Kit (Biouniquer Technology Co., Nanjing, China). A549 cells were transfected with either control or BAX siRNA for 24 h and then treated with doxorubicin for another 24 h. Cells were harvested through trypsinization and washed two times with cold PBS. Cells were centrifuged at 3000 r/min for 5 min, then the supernatant was discarded and the pellet was resuspended in 500 *μ*l 1 × Annexin V binding buffer in a 1.5 ml culture tube and later incubated with 5 *μ*l of FITC-conjugated Annexin V and 5 *μ*l of PI for 10 min at room temperature in the dark. The samples were analyzed with FACS using the Cell Quest Research software (Beckman Coulter, Brea, CA, USA).

### Statistical analysis

All data were presented as the mean±S.E.M. Statistical analysis for RT-PCR was determined by Student's *t*-test, while others were determined by one-way analysis of variance with Bonferroni's multiple comparison *post hoc test* for comparisons of more than two groups and a two-tailed Student's *t*-test if two groups were compared. *A P-*value of <0.05 was considered statistically significant and is indicated as: *P<0 . 05, **P<0 . 01 and ***P<0 . 001.
