# Supplementary material for: DRAM1 regulates apoptosis through increasing protein levels and lysosomal localization of BAX
Source: Cell Death Dis. 2015 Jan 29;6(1):e1624–. doi: 10.1038/cddis.2014.546 (PMC4669745; doi:10.1038/cddis.2014.546)
Supplement: Supplementary Figure Legends [file cddis2014546x1.doc]

**Figure 1** DRAM1 increases BAX protein levels independent of transcription. (**a**) A549 cells were treated with 3NP (500 μM) and harvested 24, 48 and 72 h later. Bars represent mean ± SE; n = 4; **P< 0.01 vs 0 h; ##P<0.01 vs 0 h. (**b**) A549 cells were transfected with DRAM1 siRNA or a nonsilencing siRNA for 24 h. Cells were then treated with or without 3NP (500 μM) for another 24 h. Bars represent mean ± SE; n = 4; **P< 0.01 vs NC; #P< 0.05, ##P< 0.01 vs NC; $$P< 0.01 vs NC+3NP; &&P< 0.01 vs NC+3NP. (**c**) Hela cells were transfected with vector or DRAM1 plasmid for 48 h. Bars represent mean ± SE; n = 4; **P< 0.01 vs vector; ##P< 0.01 vs vector. (**d**) Hela cells were treated with 3NP (500 μM) for 48 h. RNAs were isolated and amplified with qRT-PCR. Bars represent mean ± SE; n = 4; **P< 0.01 vs control. (**e**) Hela cells were transfected with vector or DRAM1 plasmid for 48 h. RNAs were isolated and amplified with qRT-PCR. Bars represent mean ± SE; n = 4; ***P< 0.001 vs vector.

**Figure 2** BAX is degraded by autophagy and DRAM1 interferes with BAX degradation. (**a** and **b**) Cells were treated with UPS inhibitor MG132 (40 μM) for 1, 2, 4 and 6 h or lactacystin (4 μM) for 2, 4, 6, and 9 h. The cell lysates were subjected to immunoblotting. β-actin was served as a loading control. Bcl-2 was served as a positive control. Bars represent mean ± SE; n = 3. (**c**) Hela cells were treated with autophagy inhibitor 3MA (1.5 mg/ml) for 1, 2, 4 and 6 h. Bars represent mean ± SE; n = 4; *P<0.05, **P<0.01 vs 0h. (**d**) Hela cells were treated with autophagy inhibitor chloroquine (20 μM) for 2, 4, 6 and 12 h. Bars represent mean ± SE; n = 4; *P<0.05, **P<0.01 vs 0h. (**e**) Cells were transfected with Atg5 siRNA for 48 h to inhibit autophagy. Bars represent mean ± SE; n=4; *P<0.05, **P<0.01 vs NC.

**Figure 3** DRAM1 interacts with BAX. (**a**) Cell lysates were immunoprecipitated (IP) with an anti-BAX antibody, then immunoblotted (IB) against DRAM1. (**b**) Cell lysates were immunoprecipitated (IP) with an anti-DRAM1 antibody, then immunoblotted (IB) against BAX. (**c**) Cell lysates from cells transfected with vector or DRAM1-pcDNA4 were IP with an anti-BAX antibody and then IB against DRAM1. (**d**) Cell lysates as mentioned in panel **c** were IP with an anti-DRAM1 antibody and then IB against BAX. (**e**) The half-life of BAX was prolonged in DRAM1 overexpressing cells. A549 cells and those stably transfected with DRAM1 were treated with cycloheximide (50 μg/ml). Cells were harvested at 0, 2, 4, 6, 8, 12 and 24 h after treatment. Bars represent mean ± SE; n = 3. The density of immunoreactivity for each band were measured and normalized to the density at t = 0 (100%). The log10 of the percentage of density was plotted versus time, and the t1⁄2 was calculated from the log10 of 50%.

**Figure 4** BAX translocates to lysosome, releases cathepsin B and activates the BID cleavage. (**a**) A549 cells were treated with 3NP (500 μM) for 48 h. The cytoplasmic (Cyto) and lysosomal (Lyso) were fractionated, and then subjected to SDS-PAGE. (**b**) Lysosomes from cells transfected with or without DRAM1 siRNA were fractionated, and then incubated with BAX protein at 37 °C for 2 h. The lysosomal fractions were subjected to SDS-PAGE. (**c**) A549 cells were treated with 3NP (500 μM) for 48 h. The cytoplasmic (Cyto) and lysosomal (Lyso) were fractionated, and then subjected to SDS-PAGE. (**d**) A549 cells were transfected with BAX siRNA or a nonsilencing siRNA for 24 h, and cells were treated with 3NP (500 μM) for another 24 h, cytoplasm were fractionated for Western blot analysis. (**e**) A549 cells were transfected with BAX siRNA or a nonsilencing siRNA for 24 h, then treated with or without 3NP (500 μM) for another 24 h. Bars represent mean ± SE; n = 4; **P < 0.01 vs NC; ##P< 0.01 vs 3NP+NC. (**f**) A549 cells were treated with 3NP (500 μM) and the cathepsin B inhibitor for 24 h. Bars represent mean ± SE; n = 4; **P < 0.01 vs control; ##P< 0.01 vs 3NP.

**Figure 5** BAX mediates the pro-apoptotic role of DRAM1. (**a**) A549 cells were treated with 3NP (500 μM) and harvested 24, 48 and 72 h late for preparation of mitochondrial and cytosolic fractions. Bars represent mean ± SE; n = 3; **P < 0.01 vs 0h. (**b**) A549 cells were transfected with DRAM1 siRNA or Non-sil siRNA for 24 h and then treated with 3NP (500 μM) for 24 h. Bars represent mean ± SE; n = 3, **P < 0.01 vs NC; #P<0.05, ##P< 0.01 vs 3NP+NC. (**c**) A549 cells were transfected with BAX siRNA or Non-sil siRNA for 24 h and then treated with 3NP (500 μM) for 24 h. Bars represent mean ± SE; n = 3, **P < 0.01 vs NC; ##P< 0.01 vs 3NP+NC.

**Figure 6** BAX mediates pro-apoptotic role of DRAM1 partially through tBID. (**a**) Immunoblot analysis of activation of caspase-3. A549 cells were treated with 3NP (500 μM) for 24, 48 and 72 h, the activation of caspase-3 was determined with immunoblotting. Bars represent mean ± SE; n = 3; *P<0.05, **P <0.01 vs 0 h. (**b**) A549 cells were transfected with DRAM1 siRNA or Non-sil siRNA for 24 h and then treated with 3NP (500 μM) for 24 h. Bars represent mean ± SE; n = 3, **P < 0.01 vs NC; #P<0.05, ##P< 0.01 vs 3NP+NC. (**c**) A549 cells were transfected with BAX siRNA or Non-sil siRNA for 24 h and then treated with 3NP (500 μM) for 24 h. Bars represent mean ± SE; n = 3, **P < 0.01 vs NC; ##P< 0.01 vs 3NP+NC. (**d**) Efficiency of siRNA-mediated downregulation of BID. (**e**) A549 cells were transfected with BID siRNA or Non-sil siRNA for 24 h and then treated with 3NP (500 μM) for another 24 h. Active caspase-3 was determined with immunoblotting. Bars represent mean ± SE; n = 3; *P<0.05 vs NC, #P <0.05vs NC+3NP.

**Figure 7** DRAM1 regulates cell death through BAX. (**a**) A549 cells were treated with 3NP (500 μM) for 24, 48 and 72 h, and cell viability was evaluated. Bars represent mean ± SE; n = 4; **P< 0.01, ***P < 0.001 vs oh. (**b**) Cell viability was evaluated after transfection of cells with DRAM1 siRNA in the presence or absence of 3NP (500 μM). Bars represent mean ± SE; n = 4; **P< 0.01 vs NC, ##P< 0.01vs NC+3NP. (**c**) Cell viability was evaluated after transfection of cells with BAX-pcDNA4 or BAX siRNA in the presence or absence of 3NP (500 μM). Bars represent mean ± SE; n = 4; **P< 0.01 vs NC; #P< 0.05 vs NC+3NP; $$P< 0.01 vs NC+3NP. (**d**) A549 cells were transfected with BID siRNA for 24 h, then treated with 3NP (500 μM) for another 24 h. Bars represent mean ± SE; n = 3; **P< 0.01 vs NC; #P<0.05 vs 3NP+Non-sil siRNA. (**e**) A549 cells were transfected with BAX siRNA or Non-sil siRNA for 24 h and then treated with 3NP (500 μM) for another 24 h. After treatment, apoptotic cells were determined with FACS after Annexin V-FITC and PI staining.

**Figure 8** Proposed model for the action of DRAM1 in 3NP- and doxorubicin-induced autophagy and apoptosis.

**Supplementary Figure 1** DRAM1 increases BAX protein. (**a**) Hela cells were treated with 3NP (500 μM) and harvested 24 and 48 h later. Bars represent mean ± SE; n = 4; *P<0.05 vs 0 h. (**b**) A549 cells were treated with doxorubicin (5 μg/ml) for 12 and 24 h. Bars represent mean ± SE; n = 3; *P<0.05 vs 0 h. (**c**) Hela cells were treated with doxorubicin (5 μg/ml) for 12, 24 and 36 h. Bars represent mean ± SE; n = 3; **P<0.01 vs control, ##P<0.01 vs 0 h. (**d**) A549 cells were transfected with DRAM1 siRNA or a nonsilencing siRNA for 24 h, and then treated with doxorubicin (5 μg/ml) for another 24 h. Bars represent mean ± SE; n = 3; **P<0.01 vs NC; #P<0.05 vs NC; $P<0.05, $$P<0.01 vs NC+doxorubicin; &&P<0.01 vs NC+doxorubicin. (**e**) Hela cells were transfected with vector or BAX plasmid for 48 h. Bars represent mean ± SE; n = 3; ns (no significance) vs vector.

**Supplementary Figure 2** BAX is degraded by autophagy. (**a**) Hela cells were treated with MG132 (40 μM) for 4 h. MG132 increased the accumulation of ubiquitinated proteins. (**b**) Hela cells were treated with the autophagy inhibitor chloroquine (20 μM) or bafilomycin (100 nM) for 6 h. Whole-cell lysates Western blot analysis showed that both chloroquine and bafilomycin increased the accumulation of p62 and LC3-II. (**c** and **d**) A549 cells were treated with autophagy inhibitor chloroquine (20 μM) for 2, 4, 6 and 12 h or 3MA (1.5 mg/ml) for 1, 2, 4 and 6 h. Bars represent mean ± SE; n = 3; *P<0.05 vs 0 h, **P<0.01 vs 0 h. (**e** and **f**) HCT116 cells were treated with autophagy inhibitor chloroquine (20 μM) for 2, 4, 6 and 12 h or 3MA (1.5 mg/ml) for 1, 2, 4 and 6 h. Bars represent mean ± SE; n = 3; *P<0.05 vs 0 h, **P<0.01 vs 0 h.

**Supplementary Figure 3** DRAM1 interferes with BAX degradation. (**a** and **b**) Autophagy activation accelerated the degradation of BAX in the condition of DRAM1 suppression. A549 cells and DRAM1 knocked-down cells were treated with rapamycin for 6, 12, 24, 36 and 48 h. Bars represent mean ± SE; n = 4; A549 group: **P<0.01, ***P<0.001 vs 0 h; #P<0.05, ##P<0.01, ###P<0.001 vs 0 h; A549/DRAM1 KD group: #P<0.05, ##P<0.01 vs 0 h. (**c** and **d**) Hela cells transfected with DRAM1-pcDNA4 was treated with or without autophagy inhibitor 3MA for 4 h (**c**) or chloroquine for 6 h (**d**). Cells were harvested and protein levels of DRAM1 and BAX were analyzed with immunoblotting. Bars represent mean ± SE; n = 4; *P<0.05, **P<0.01,***P<0.001 vs control.

**Supplementary Figure 4** DRAM1 interacts with BAX. (**a**) A549 cells were transfected with or without DRAM1-pCDNA4. Cell lysates were incubated with GST or GST-BAX protein for 2 h, and then pull down. The cell lysates and bound proteins were subjected to SDS-PAGE and immunoblot analysis. (**b**) Cells transfected with vector or DRAM1-pcDNA4 were subjected to immunoprecipitation (IP) with an anti-BAX antibody and then immunoblotting (IB) against DRAM1 and Bcl-2. (**c**) HeLa cells were transferred to serum-free medium for 4 h. Cell lysates were subjected to IP with an anti-DRAM1 antibody and then IB against BAX.

**Supplementary Figure 5** BAX translocates to lysosomes. (**a**) A549 cells were transfected with EGFP-BAX, Non-sil siRNA or DRAM1 siRNA for 24 h. Cells were then treated with or without 3NP (500 μM) for another 24 h. Cells were incubated with LysoTracker (0.5 μM), co-localization of EGFP- BAX (green) and the LysoTracker (red) was assessed. (**b**) A549 cells were transfected with Vector or DRAM1-pcDNA4 for 24 h. Cells were then treated with or without 3NP (500 μM) for another 24 h for preparation of mitochondrial, lysosomal and cytosolic fractions. The subcellular fractions were subjected to SDS-PAGE. (**c**) A549 cells were treated with or without 3NP (500 μM) for 24 h. The co-localization of Lamp2 (red) and cathepsin B (green) was assessed.

**Supplementary Figure** **6** BAX mediates pro-apoptotic role of DRAM1 partially through tBID. (**a**) Efficiency of siRNA-mediated downregulation of BAX. Bars represent mean ± SE; n = 4; **P<0.01 vs NC; ##P<0.01 vs NC; $$P<0.01 vs 3NP+Non-sil siRNA. (**b**) Cells treated with 3NP were transfected with non-sil siRNA or with BAX siRNA and then lysosomes were fractionated, the integrity of each sample were tested. Bars represent mean ± SE; n = 4; *P<0.05 vs Non sil-RNA; #P<0.05 vs 3NP+Non-sil siRNA. (**c**) Lysosomes were isolated and then incubated with or without BAX protein at 37 °C for 2 h. The supernatant and lysosomal fractions were subjected to SDS-PAGE and immunoblotting for the indicated proteins. (**d**) A549 cells were treated with 3NP (500 μM) and the caspase 8 inhibitor zIETD for 24 h. Cells were harvested and protein levels of BID were analyzed with immunoblotting. Bars represent mean ± SE; n = 4; **P < 0.01 vs control. (**e**) A549 cells were treated with etoposide (50 μM) for 12 and 24 h. (**f**) A549 cells were treated with etoposide (50 μM) and the caspase 8 inhibitor zIETD for 24 h. Cells were harvested and protein levels of BID were analyzed with immunoblotting. Bars represent mean ± SE; n = 3; **P < 0.01 vs control; #P < 0.05 vs etoposide.

**Supplementary Figure 7** Knockdown of DRAM1 inhibits the 3NP- induced apoptosis and increases cell viability. (**a**) A549 cells were transfected with vector or DRAM1 plasmid for 48 h. Whole-cell lysates Western blot showed that overexpression DRAM1 failed to induce caspase-3 activation. (**b**) A549 cells and Hela cells were transfected with Vector or DRAM1 plasmid for 48 h. After 3NP treatment, apoptotic cells were determined with FACS after Annexin V-FITC and PI staining. (**c**) A549 cells were knockdown of DRAM1 for 24 h and then treated with 3NP for 24 h. The cells were then processed to measure levels of active caspase-3/caspase-8 uing the Caspase-Glo kit. Bars represent mean ± SE; n = 4; **P< 0.01 vs NC, ##P< 0.01vs NC+3NP. (**d**) Cell viability was evaluated after transfection of cells with DRAM1-pcDNA4 in the presence or absence of 3NP (500 μM). Bars represent mean ± SE; n = 3; **P< 0.01 vs NC, ##P< 0.01vs NC+3NP. (**e**) Cell viability was evaluated after transfection of cells with Atg5 siRNA in the presence or absence of 3NP (500 μM). Bars represent mean ± SE; n = 4; **P< 0.01 vs NC, ##P< 0.01vs NC+3NP.
