# Supplementary figures and images for: DRAM1 regulates apoptosis through increasing protein levels and lysosomal localization of BAX
Source: Cell Death Dis. 2015 Jan 29;6(1):e1624–. doi: 10.1038/cddis.2014.546 (PMC4669745; doi:10.1038/cddis.2014.546)

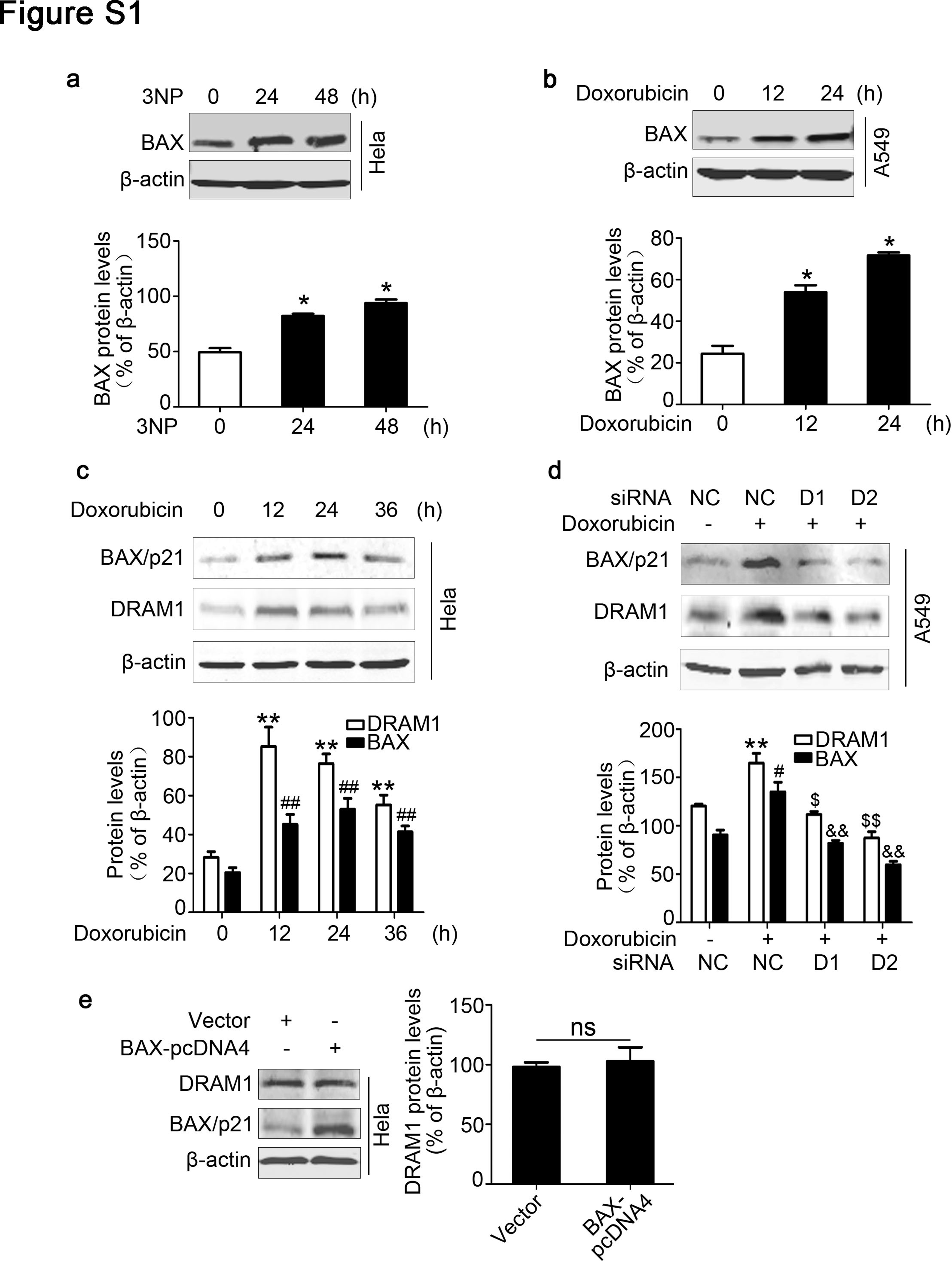

Supplement: Supplementary Figure S1 [file cddis2014546x2.tif]

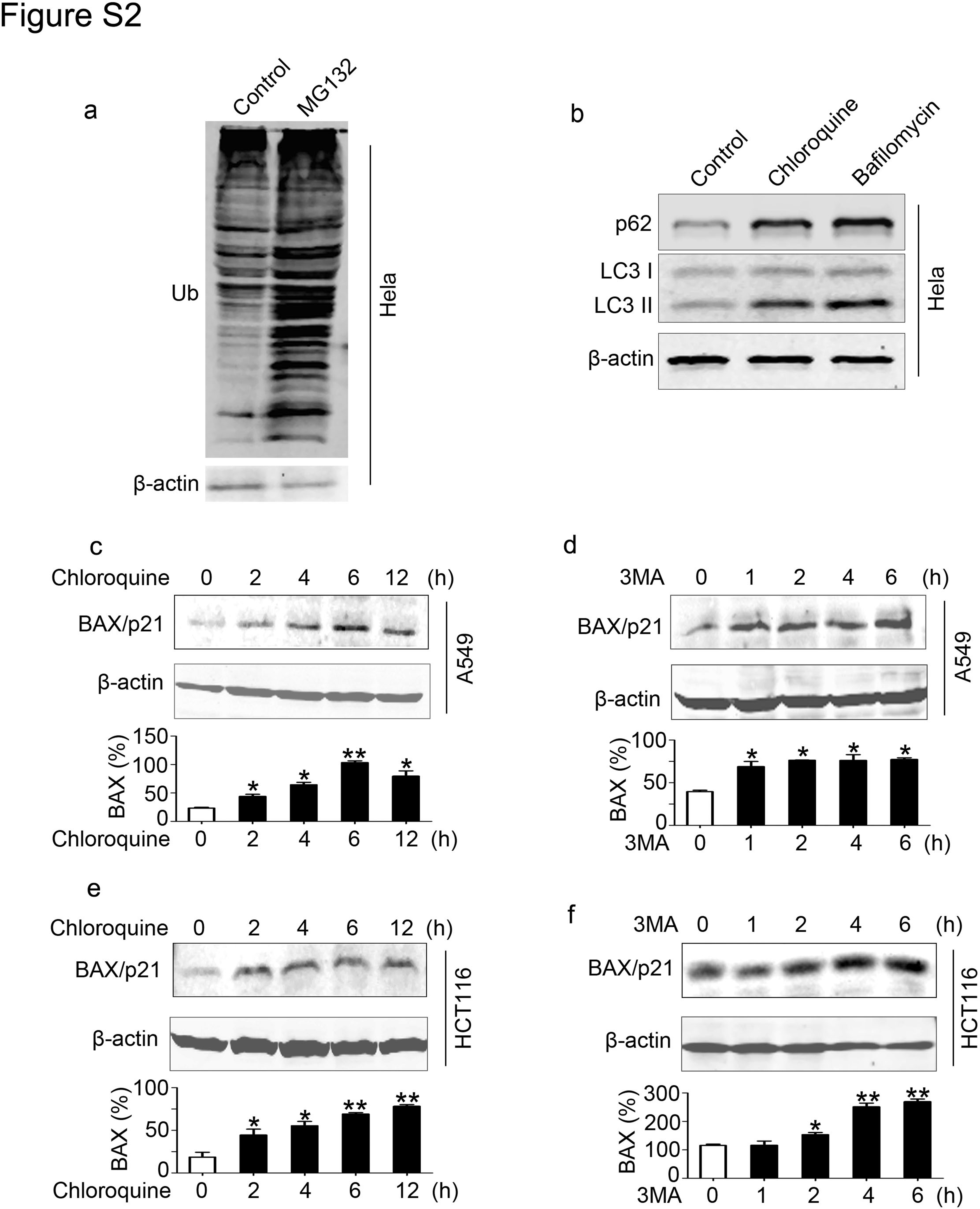

Supplement: Supplementary Figure S2 [file cddis2014546x3.tif]

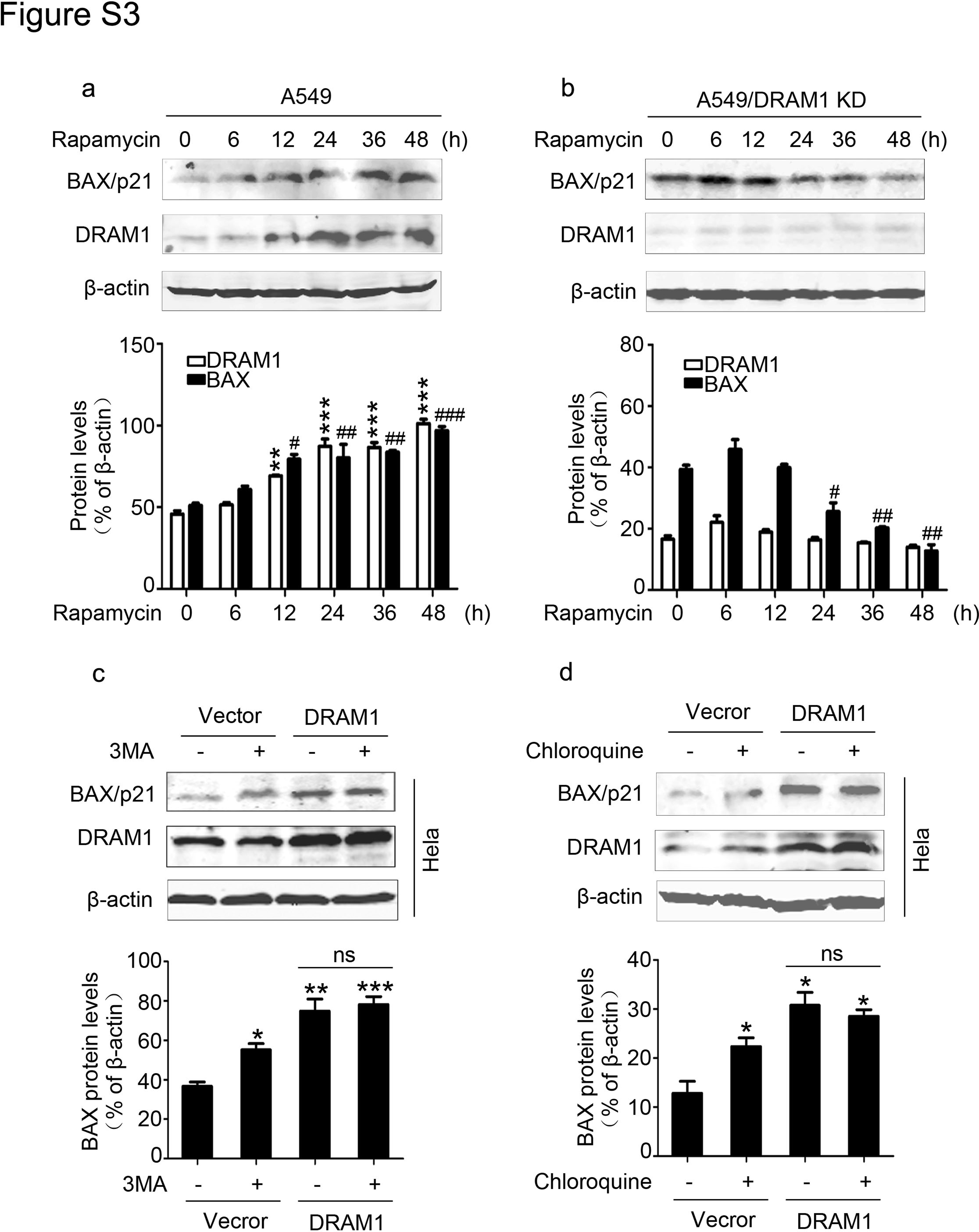

Supplement: Supplementary Figure S3 [file cddis2014546x4.tif]

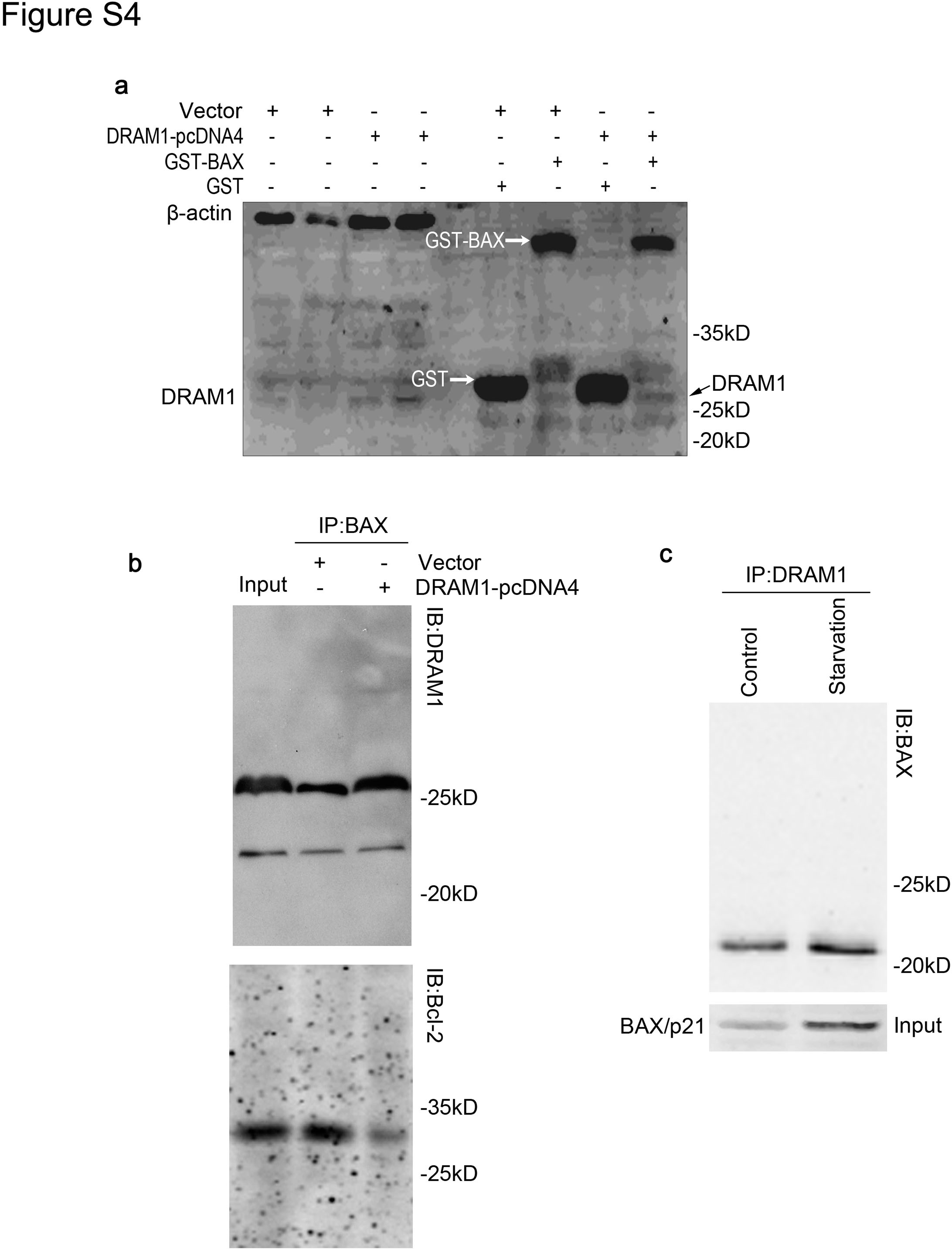

Supplement: Supplementary Figure S4 [file cddis2014546x5.tif]

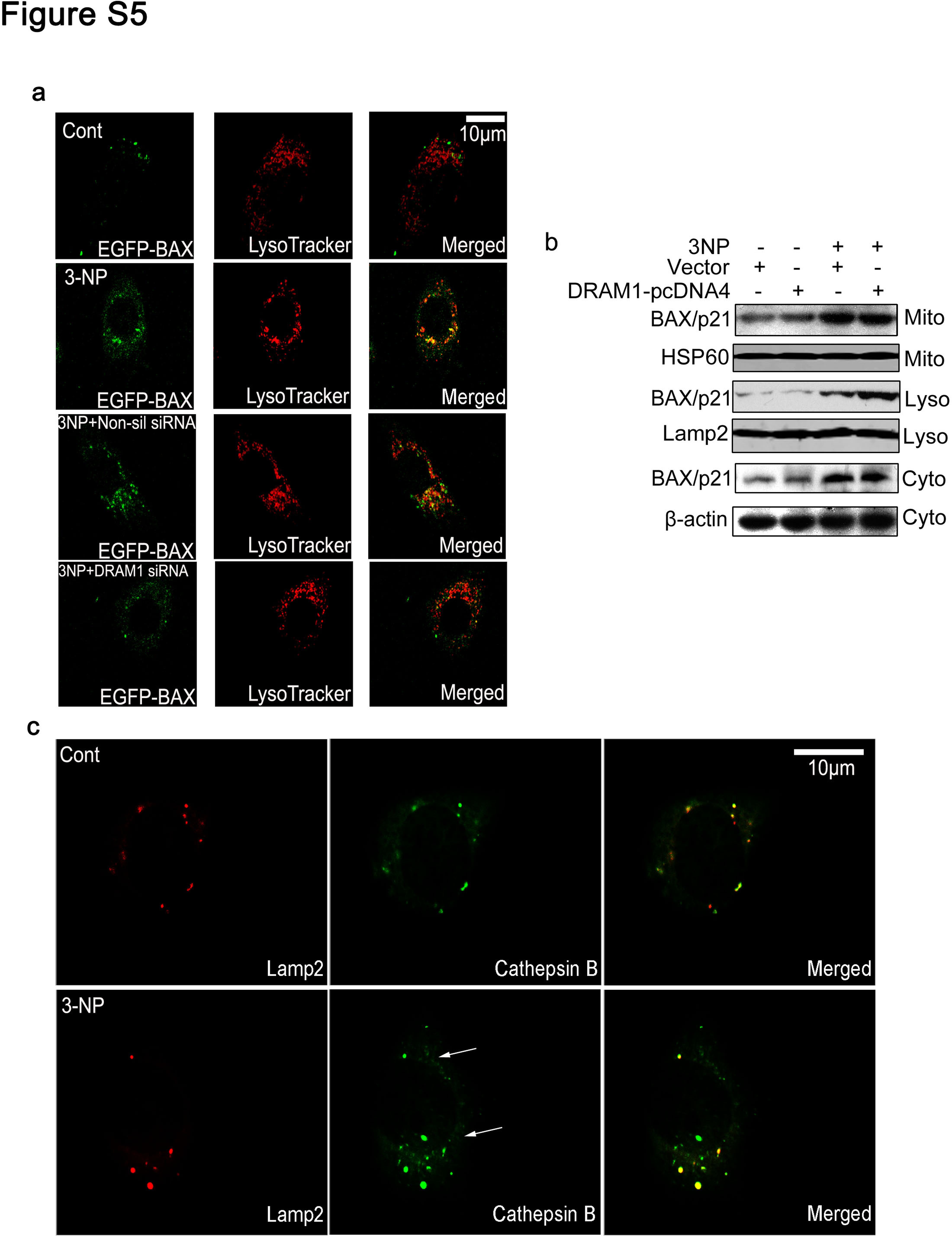

Supplement: Supplementary Figure S5 [file cddis2014546x6.tif]

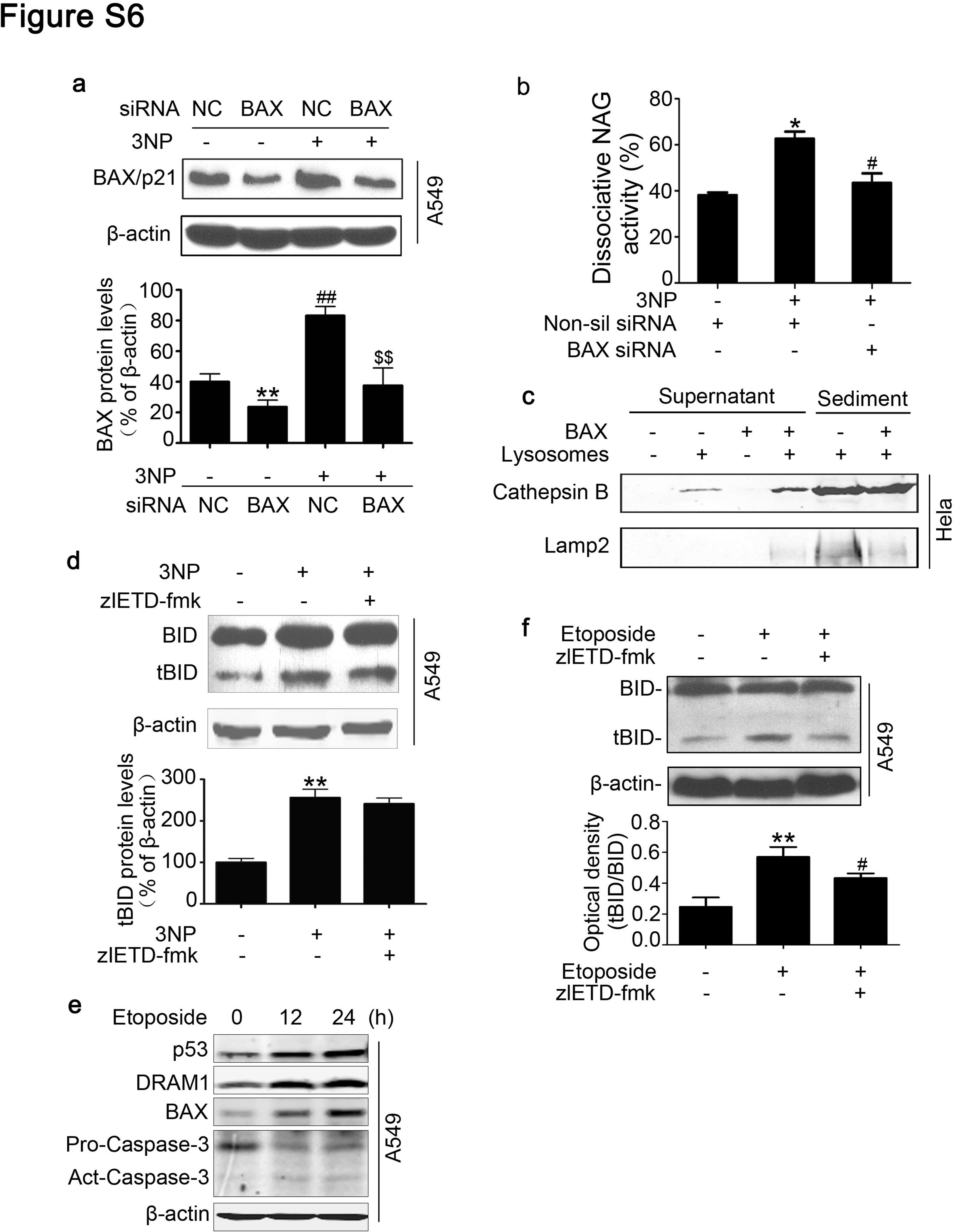

Supplement: Supplementary Figure S6 [file cddis2014546x7.tif]

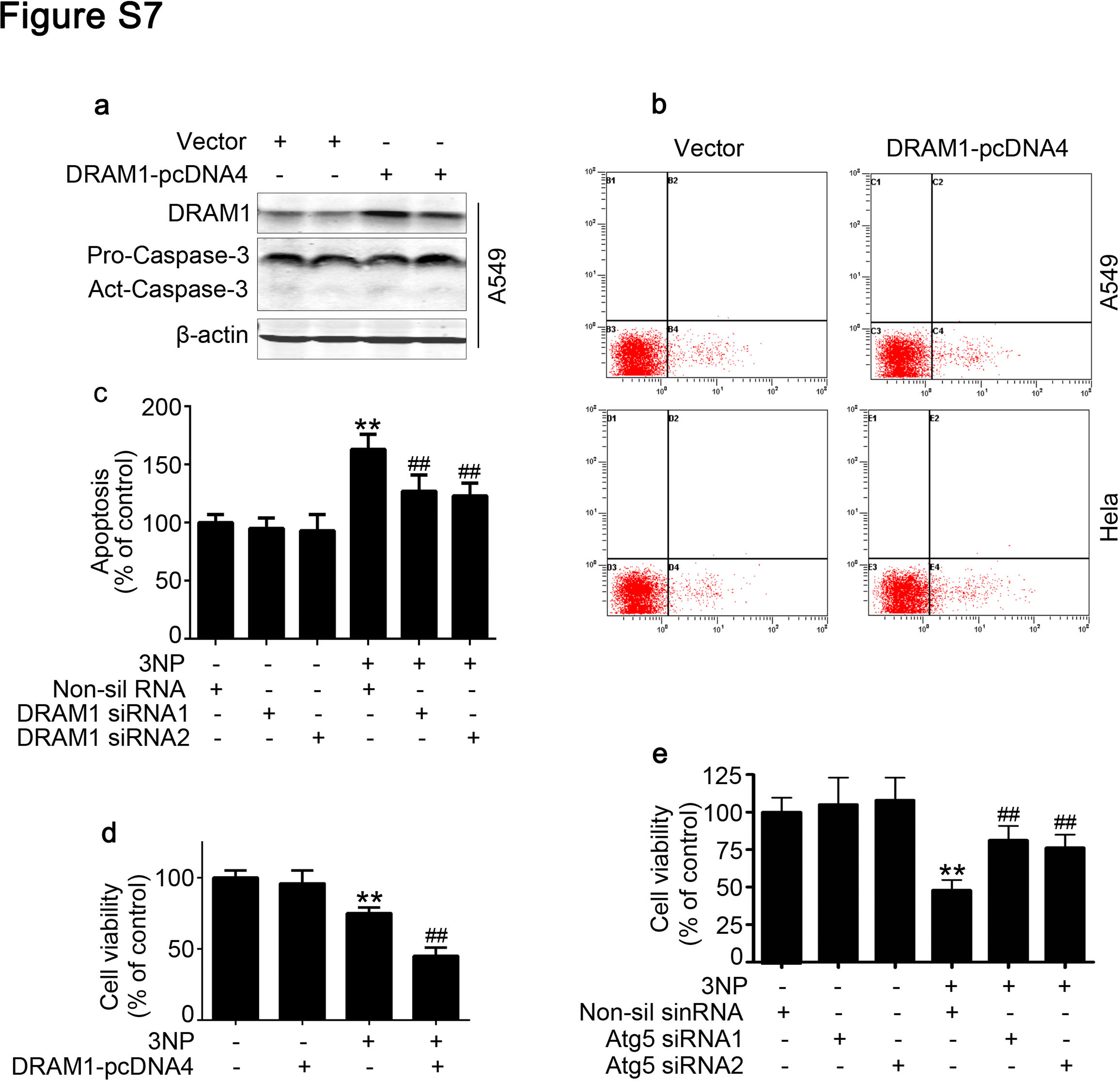

Supplement: Supplementary Figure S7 [file cddis2014546x8.tif]
